# In Vivo Degradation and Local Tissue Response of Experimental Carp Collagen Membranes: Micro‐MRI and Histological Analysis

**DOI:** 10.1002/bip.70045

**Published:** 2025-08-18

**Authors:** Michele Bujda, Tomáš Suchý, Vít Herynek, Jaroslava Dušková, Margit Žaloudková, Luděk Šefc, Karel Klíma, Robert Plachý, René Foltán

**Affiliations:** ^1^ First Faculty of Medicine Charles University Prague Czech Republic; ^2^ Department of Oral and Maxillofacial Surgery General University Hospital in Prague Prague Czech Republic; ^3^ Department of Composites and Carbon Materials, Institute of Rock Structure and Mechanics Czech Academy of Sciences Prague Czech Republic; ^4^ Center for Advanced Preclinical Imaging (CAPI) First Faculty of Medicine, Charles University Prague Czech Republic; ^5^ Institute of Pathology Charles University Prague Czech Republic; ^6^ Faculty of Medicine in Pilsen Charles University Pilsen Czech Republic

**Keywords:** carp skin collagen, GBR membrane, guided bone regeneration, in vivo collagen degradation, inflammatory reaction, micro‐MRI analysis, TERM

## Abstract

Collagen membranes are widely used in tissue and bone engineering, including guided bone regeneration (GBR). For effective and uninterrupted bone healing, a GBR membrane must maintain its functionality for an initial critical period of 4 weeks. A novel carp collagen sponge has already shown promise as a wound coating and vascular graft coating, making it a candidate for GBR applications as well. To enhance the mechanical properties and longevity of GBR membranes, we modified the basic carp collagen membrane with combinations of l‐lactide, ε‐caprolactone, d,l‐lactide, and glycolide in various molar ratios. While traditional methods rely on histological evaluation to assess the degradation pattern and therefore suitability of GBR membranes ex vivo*,* this study employed micro‐MRI as an innovative, noninvasive approach to monitor the in vivo degradation of carp collagen membrane and its polymer‐modified variants. Our findings demonstrated that micro‐MRI is a reliable and effective method for visualizing collagen membrane degradation in vivo, up to scaffold disintegration. Among the variants tested, collagen GBR membrane coated with d,l‐lactide and glycolide in a 50:50 M ratio emerged as the most suitable for GBR purposes. However, since this study was conducted in the subcutaneous tissue of a rat model, further research is required to determine the behavior of carp collagen GBR membrane variants on bony surfaces.

## Graphical abstract

Experimental design showing preparation of carp collagen membranes, SEM visualization, implantation in rats, MRI‐based degradation monitoring, and histological assessment of local tissue response.

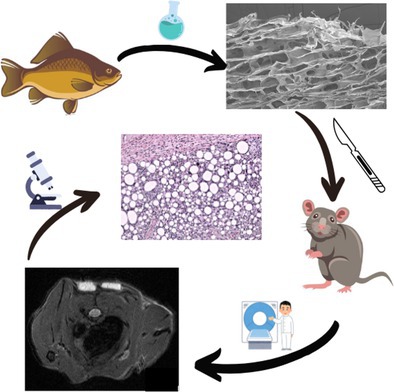

## 1 Introduction

Tissue engineering and regenerative medicine (TERM) heavily depend on advanced biomaterials capable of mimicking the extracellular matrix (ECM), promoting tissue repair and integrating with host tissues. Developing an ideal scaffold remains challenging; it requires balancing mechanical strength, degradation rate, and biocompatibility to ensure optimal performance in various TERM applications [1, 2].

Collagen‐based membranes have gained widespread use in TERM due to their exceptional biocompatibility, biodegradability, low immunogenicity, and ability to support cell adhesion, proliferation, and differentiation. These properties make collagen a versatile material for both clinical regenerative medicine and cell culture research [3]. Collagen is the most abundant structural protein in vertebrates, forming the ECM of connective tissues, that is, bone, cartilage, dentin, and cementum of teeth, and fibrous tissue [4]. It can be processed into various structural forms (e.g., membranes, sponges, gels) that are useful for specific TERM procedures.

Animal‐derived collagen, particularly, from bovine and porcine sources, remains the most common choice for tissue engineering due to its availability, cost‐effectiveness, and compatibility with human tissues. Recently, marine and fish‐derived collagen has gained attention for its lower immunogenic potential, reduced risk of disease transmission, sustainability, and lack of cultural or religious concerns [3, 5].

A novel collagen sponge derived from European carp ( *Cyprinus carpio* ) skin has demonstrated potential in drug‐eluting scaffolds for wound healing and vascular graft coatings [6, 7, 8]. This material offers modifiable properties, such as degradation rate and porosity, which can be adjusted during fabrication. However, there is a lack of research investigating its use in bone regeneration with a focus on guided bone regeneration (GBR). For GBR applications, this sponge would serve primarily as a GBR membrane and osteoconductive scaffold as we suggested in our review [9]. The proper GBR membrane must be able to immobilize the graft material, maintain space, and provide mechanical support for a minimum of 4 weeks to facilitate initial bone formation [10, 11].

An important challenge in GBR membrane design is to achieve the desired degradation rate and sufficient mechanical stability simultaneously. In the case of the carp collagen sponge, both can be modified by the optimal degree of collagen cross‐linking during preparation [12]. The next crucial step is to find a balance between the porosity and the mechanical strength of the membrane. Microporous structures enhance mechanical stability, while macroporous structures are essential for the integration, vascularization, and osteogenic processes within the host tissue [9]. In the case of the used carp collagen sponge, porosity can be adjusted during fabrication by controlling the concentration of collagen dispersion and the freezing point prior to lyophilization [12]. Modified variants of carp collagen sponge (included in Patent No. CZ2019777A3), namely sandwich collagen sponge and composite collagen sponge, have improved mechanical stability and degradation time (due to their multilayered structure) but are significantly thicker, limiting their applicability in small surgical sites, such as dental bone augmentation, which is nowadays the most common clinical case of bone regeneration procedure. The large dimensions of the scaffold in such cases could impede soft tissue closure over the defect, necessitate more extensive periosteum elevation during surgery, thus disrupting blood supply, and pose manipulation issues of the product as well.

Because of this, we have chosen a simple collagen sponge for our first experimental study. To avoid possible fast degradation of such a thin collagen layer and also to enhance its mechanical properties, we propose modifying the carp collagen sponge into a dual‐sided GBR membrane. Such a GBR membrane arrangement has already shown promising results in several experiments [13, 14]. The design involves impregnating the upper layer of the GBR membrane with biodegradable polymers to enhance mechanical properties and extend the degradation time, while leaving the lower layer unmodified to maintain osteoconductivity. This construction could deliver improvement to the scaffold (i.e., sufficient membrane longevity to allow for an undisrupted initial critical 4‐week period of bone healing) without increasing its thickness. On the other hand, the addition of polymers could result in an increase in the inflammatory reaction of local tissues as well as the fabrication costs.

Polymers such as polylactic acid (PLA), polyglycolic acid (PGA), and polycaprolactone (PCL) are commonly used in TERM due to their biocompatibility, predictable degradation rates, and safe by‐products [15]. Their incorporation into GBR membranes has become increasingly popular due to their ability to modulate scaffold properties [14].

PLA polymers are well‐known for their superior biocompatibility and mechanical strength. PLA polymers can exist in various ratios of l‐ and d‐ isomeric forms. Poly‐l‐lactide exhibits a highly crystalline structure, whereas poly‐d,l‐lactide incorporates both isomers randomly, resulting in an amorphous structure. The degradation time of poly‐l‐lactide is significantly longer than that of poly‐d,l‐lactide, given their different crystallinities, water absorption, and subsequent hydrolysis [16, 17].

Polyglycolide (PGA) is synthesized from glycolic acid and exhibits the fastest degradation rate of the aforementioned polymers. For this reason, PGA is rarely used as a single polymer material in GBR membranes, as its integrity would be lost before bone regeneration is complete [14]. However, in vitro experiments showed that incorporation of PGA fibers into the collagen membrane increases the number of cells that attach to the scaffold, enhancing its bioactivity [18].

PCL degrades more slowly than PGA and PLA, due to its hydrophobic nature and the stronger ester bonds in its structure, which require enzymatic activity for degradation. On the contrary, the degradation of PGA and PLA is mainly dependent on hydrolysis, and enzymatic activity plays a secondary role. The extended degradation time of PCL makes it advantageous for long‐term TERM applications requiring prolonged structural support, such as bone regeneration. However, PCL has lower mechanical strength compared to both PGA and PLA, which could make it disadvantageous when choosing a polymer, since a good GBR membrane should have good mechanical and load‐bearing properties [19].

To optimize the properties of carp collagen sponge (*membrane A* in our experiment), we modified the upper layer with polymer combinations (see Section 2) and created variants of an experimental GBR membrane. We hypothesize that at least one of these membranes will maintain structural integrity for the critical 4‐week period required for GBR.

In vitro studies suggest that the degradation rate of carp collagen is highly predictable; however, in vivo evaluations of this specific GBR design remain unexplored. This study aims to evaluate the in vivo degradation rate and biocompatibility of the carp collagen GBR membrane and its polymer‐modified variants in a rat model, providing critical insights into their potential for clinical applications in bone regeneration and other TERM purposes.

Traditional methods for monitoring TERM scaffolds degradation often rely only on histological approaches. In this study, we employed micro‐MRI, a noninvasive imaging modality, to assess the degradation of carp collagen scaffolds over time. To our knowledge, this is one of the first studies to utilize micro‐MRI for this purpose, providing unique insights into scaffold behavior in vivo. Subsequently, complementary histological analyzes were performed to evaluate the local tissue response, including the inflammatory reaction and the integrity of residual GBR membranes.

## 2 Materials and Methods

### 2.1 Scaffold Preparation

The carp collagen used for membranes preparation was isolated from the skin of freshwater fish—European carp ( *C. carpio* ). These were provided by the Třeboň fishery (Třeboň, Czech Republic); all the fish were of food grade and were produced via controlled breeding. The procedure for the isolation of carp collagen is described in detail in the study by Lukáč et al. [20].

Collagen membranes were prepared employing the following procedure. An aqueous collagen dispersion (4 wt%) was prepared by the swelling of collagen in deionized water, homogenized using a disintegrator (10,000 rpm, 10 min) and left for 60 min at a temperature of 20°C. The final homogenization was performed using the disintegrator (6500 rpm, 10 min). The resulting dispersion was placed in separate containers with dimensions of 10 × 10 × 2 cm, frozen at −70°C for 5 h, and then lyophilized (BenchTop 4KZL, VirTis, CA, USA). A 95 wt% ethanol solution with *N*‐ethyl‐*N*′‐(3‐dimethylaminopropyl)carbodiimide (EDC) and *N*‐hydroxysuccinimide (NHS) (Sigma‐Aldrich, MI, USA) at an EDC/NHS ratio of 4/1 (wt/wt) was used for collagen cross‐linking (24 h). 0.1 M Na_2_HPO_4_ (2 × 30 min) and deionized water (30 min) were used for the washing of the membranes. Finally, the membranes were frozen (−30°C) and lyophilized.

This basic scaffold was used for the production of all experimental GBR membranes: one without further modifications (*membrane A*) and three other designs. Corbion polymers (Purasorb) were used for their production.

### 2.2 Experimental GBR Membranes

– *Membrane A*: carp collagen scaffold
– *Membrane B*: carp collagen scaffold with superficial layer composed of copolymer of poly‐l‐lactide and PCL in a 70:30 ratio (Purasorb PLC 7015)
– *Membrane C*: carp collagen scaffold with superficial layer composed of copolymer of poly‐d,l‐lactide and glycolide in a 50:50 ratio (Purasorb PDLG 5002)
– *Membrane D*: carp collagen scaffold with superficial layer composed of copolymer of poly‐d,l‐lactide and glycolide in a 75:25 ratio (Purasorb PDLG 7502)

All polymers were dissolved in chloroform (Penta, Prague, CZ). Solutions with a polymer concentration of 10% wt were applied to the surface of the scaffolds by application with a sterile brush (approximately 5 mL of the solution was applied to a 10 × 10 cm area). The samples with the applied layer were left in the laminar box until the solvent was completely evaporated (reaching a constant weight). All experimental membranes were packed and sealed in indicator bags and sterilized using gamma irradiation (nominal dose of 25 kGy, BIOSTER, Veverská Bítýška, CZ). During surgery, each membrane was cut to the desired size of 8 × 6 × 2 mm under sterile conditions.

No dyes were used during membranes preparation or handling (surgery itself), which would simplify implant localization during in vivo imaging and histological analysis, as it could change the degradation dynamics of scaffolds.

### 2.3 Scanning Electron Microscopy

The collagen membranes were examined by scanning electron microscopy (SEM) in the high vacuum mode (STEM Apreo S2 microscope, Thermo Fisher Scientific, Waltham, MA, USA) and in the secondary electron mode at 5 and 10 keV (HV mode, Everhart–Thornley detector). Samples were sputter‐coated with Pt in an Ar atmosphere (Leica EM ACE600, Specion s.r.o., Prague, Czech Republic). The pore size and thickness of the superficial polymer layers were measured using ImageJ 1.4v software [21]. The manual mode of the ImageJ analyzer was used for measuring the average diameter of the pores (*n =* 200); at least 40 pores were assessed on each of the five SEM micrographs (mag. ×500 and ×1000). Randomly selected pores were analyzed for both the long and short pore axes. The thickness of the superficial layers was measured similarly from five SEM micrographs (mag. ×1000 and ×2000, *n = 40*).

### 2.4 Experimental Design and Surgical Approach

Twelve‐week‐old male Wistar rats (provided by Velaz, s.r.o, Prague, Czech Republic) were used as the animal model. The animals were housed at the Center for Advanced Preclinical Imaging (CAPI), Prague, where surgeries and micro‐MRI visualization were performed.

All procedures complied with Act No. 246/1992 Coll. on the Protection of Animals Against Cruelty, and the experimental protocol was approved by the Ethics Committee of the First Faculty of Medicine, Charles University, and the Ministry of Education, Youth and Sports of the Czech Republic (Approval No. MSMT‐26149/2022‐4). The experiments followed Directive 2010/63/EU on the protection of animals used for scientific purposes.

A total of 21 rats were randomly divided into three groups (*n* = 7 per group). Each animal underwent surgery under general anesthesia induced by 3% isoflurane in air delivered through an enclosed chamber. After sedation, the rat was transferred to the heated operating table and steadily secured. Maintenance of anesthesia was achieved using 1.5%–2% isoflurane administered through a rodent face mask. Each rat was properly marked and given a specific identification number in the form of *x*.*y*, where *x* was the number of the group (1 for 4 weeks, 2 for 12 weeks, and 3 for 16 weeks) and *y* was the number of the particular rat in each group, that is, 1–7.

The surgical procedure was performed under sterile conditions. The surgical site on the back of the rat was shaved and disinfected with Skinsept Mucosa (Ecolab Deutschland GmbH, Germany). Four separate 1 cm‐long incisions were made, and subcutaneous pockets were carefully created to prevent the merging of the individual spaces. *Membranes A, B, C*, and *D* of sizes 8 × 6 × 2 mm (in dry state) were hydrated in normal saline (B. Braun, Melsungen AG, Germany) before implantation. As a negative control, another skin incision with subcutaneous dissection, but without membrane implantation, was made. All five incisions were sutured with nonresorbable intradermal suture with buried knots to prevent the animals from biting them off.

Postoperative care included subcutaneous administration of the analgesic, ketoprofenum (Ketonal, Sandoz Pharmaceuticals d.d., Ljubljana, Slovenia), in a dose 5 mg/kg. After surgery, each individual animal was transferred from the operating table back to the corresponding cage and allowed to calmly recover from anesthesia. None of the animals experienced serious illness, signs of inflammation or necrosis of the operation site, or death. None of the animals was excluded from the experiment postoperatively. The animals were euthanized at predefined intervals: Group 1 at 4 weeks, Group 2 at 12 weeks, and Group 3 at 16 weeks.

### 2.5 Assessment

#### 2.5.1 Micro‐MRI

In every rat, in vivo visualization of GBR membranes was performed the day after surgery (Day 1) and then at specific time intervals for each group. In Group 1: 7, 14, 28 days after surgery; in Group 2: 7, 14, 28 days, 8 weeks (56 days), and 12 weeks (84 days) after surgery; in Group 3: 7 days, 14, 28 days, 8, 12, and 16 weeks (112 days) after surgery.

MRI was performed under general inhalation anesthesia induced by spontaneous inhalation of isoflurane in an air solution (3% isoflurane for induction and 1.5%–2% for maintaining anesthesia) delivered through a rodent face mask.

MRI was performed using an animal 7 T scanner (MR Solutions, Guildford, UK) equipped with a ^1^H rat whole‐body volume coil. Implanted GBR membranes were scanned by T1‐weighted turbospin echo sequences in the axial and coronal directions (TE = 11 ms, TF = 4, TR = 1200 ms, NA = 2 in the coronal and NA = 4 in the axial directions, fat suppression, matrix 256 × 256, slice thickness 1 mm, FOV = 60 × 60 mm^2^) as well as by T2‐weighted turbospin echo sequences with the same geometry (effective TE = 40 ms, turbofactor TF = 8, TR = 2500 ms, number of acquisitions NA = 2 in coronal and NA = 4 in the axial directions, fat suppression, matrix 256 × 256, slice thickness 1 mm, field of view FOV = 60 × 60 mm^2^). Sequences were respiratory‐triggered to minimize motion artifacts caused by breathing.

MRI scans were processed using ImageJ 1.4v software [21]. In each scan, each membrane was segmented in the axial plane, and its area was measured across sequential 1 mm‐thick slices. The total volume was then calculated as the sum of these areas multiplied by slice thickness, that is, 1 mm. Thresholds were adjusted based on T1‐ and T2‐weighted signal contrasts to distinguish membrane remnants from surrounding soft tissue. Given the resolution limit (1 mm slices), very small remnants may have escaped detection due to the partial volume effect. Histological analysis was used as a reference standard to validate MRI observations, particularly, where membrane presence was ambiguous on imaging.

Changes in the signal intensities in both T1‐ and T2‐weighed images and their standard deviations were also recorded and evaluated in order to refer to the structural changes of the implanted collagen GBR membranes.

#### 2.5.2 Histological Analysis

Soft tissue samples from the skin and collateral subcutaneous and muscular tissue were taken from each implantation site. Samples were properly labeled with sutures and placed in 4% buffered formaldehyde solution (Medilab, Czech Republic), kept at room temperature, and transported to the pathology laboratory (Institute of Pathology, General University Hospital in Prague), where samples were handled, processed, and evaluated as follows.

Two excisions were taken from the supplied samples and embedded in paraffin. Histological sections were taken at surgical sites and stained with hematoxylin–eosin (HE) and marked with group, rat number, and location. For each histological sample (including negative control—skin incision without implanted membrane), an irritancy score was determined. Evaluation of the inflammatory response in subcutaneous tissue was carried out using the adapted scoring system proposed by Lindner et al. to fit our modified study design. Each GBR membrane “overall irritancy score” was calculated as the difference between the particular membrane irritancy score and the control irritancy score of a particular rat. The irritancy score is the number determined by the local cell types and tissue present around the implanted membrane, namely polymorphonuclear cells, lymphocytes, plasma cells, macrophages, giant cells, necrosis, neovascularization, fibrosis, and fatty infiltrate. According to the overall irritancy score, the “irritancy status” of an experimental GBR membrane was determined. The membrane was considered a “nonirritant” in the case of an overall irritancy score of 0.0–2.9, a “slight‐irritant” for a score of 3.0–8.9, a “moderate irritant” for values of 9.0–15.0, and a “severe irritant”, if the score exceeded 15.0. In the event of a negative “overall irritancy score”, the result was considered to be 0 (i.e., nonirritant) [22]. Based on this, membranes with a score of 0 elicit the same soft tissue irritation as the surgery itself.

In the same histological sections, the thickness of the residual membranes and the newly formed fibrous layer in the implanted area (if present) were measured at each time point.

### 2.6 Statistical Analysis

Statistical analysis was performed in RStudio (version 4.4.1); the graphical visualization used packages “ggplot2” and “ggtable” [23, 24].

Data normality was assessed using histograms, Q‐Q plots, and the Shapiro–Wilk test. Outliers were identified using a modified *Z*‐score based on median and MAD (median absolute deviation). They were excluded from statistical analysis as well as graphical visualizations in case the modified *Z*‐score exceeded |3.5|. In the groups with fewer than five observations, no outlier removal was performed. Some of the boxplots presented show all remaining data points (after removal of outliers by modified *Z*‐score)—including those flagged as outliers by the conventional 1.5 × IQR rule—to transparently illustrate the variability within the residual dataset.

Due to the non‐normal distribution of the data and the decreasing number of observations over time—due to both membrane disintegration and planned euthanasia of animals at 4 and 12 weeks—we used generalized estimating equations (GEEs) for the analysis. GEE is well suited for correlated data arising from repeated measurements within the same subjects (rats) and provides robust parameter estimates even when standard normality assumptions are not fully met. An exchangeable correlation structure was specified, assuming equal correlation between repeated measurements from the same rat. The membrane type and the time point were entered as fixed effects. The estimated marginal means (EMMs) for each membrane were calculated using the “emmeans” package [25], and pairwise comparisons between membranes were made with Turkey‐adjusted contrasts. These comparisons are reported as unstandardized differences (for scaffold volumes and intensity/deviation values measured by MRI, as well as histologically determined irritancy score), along with their 95% confidence intervals (CI). All CIs in the report were calculated using *t*‐values for particular degrees of freedom, which directly reflect the magnitude of the effect on the original measurement scale. We chose not to report standardized effect sizes (e.g., Cohen's *d*), as the raw mean differences in the original measurement units (e.g., mm^3^, intensity, score) are directly interpretable and contextually meaningful. A *p*‐value < 0.05 was considered statistically significant.

The degradation half‐lives of experimental collagen membranes were derived from descriptive trends in MRI‐based volume measurements over time. No mathematical model (e.g., first‐order exponential decay) was applied, as the degradation profiles deviated from idealized kinetics. Instead, half‐lives were approximated based on the period required for each membrane type to lose approximately 50% of its initial EMM volume. These values are intended to provide a comparative, illustrative indication of scaffold persistence rather than precise kinetic parameters. No formal statistical comparison of half‐life estimates was performed.

Some of the measured parameters, that is, later time points in MRI volume and intensity measurements, data comprising residual membrane thickness and newly formed fibrous layer thickness in histological slices were too sparse for statistical analysis. In that case, only descriptive statistics and graphical visualizations were performed.

## 3 Results and Discussion

### 3.1 Membranes Preparation and Structural Characterization

**FIGURE 1:**
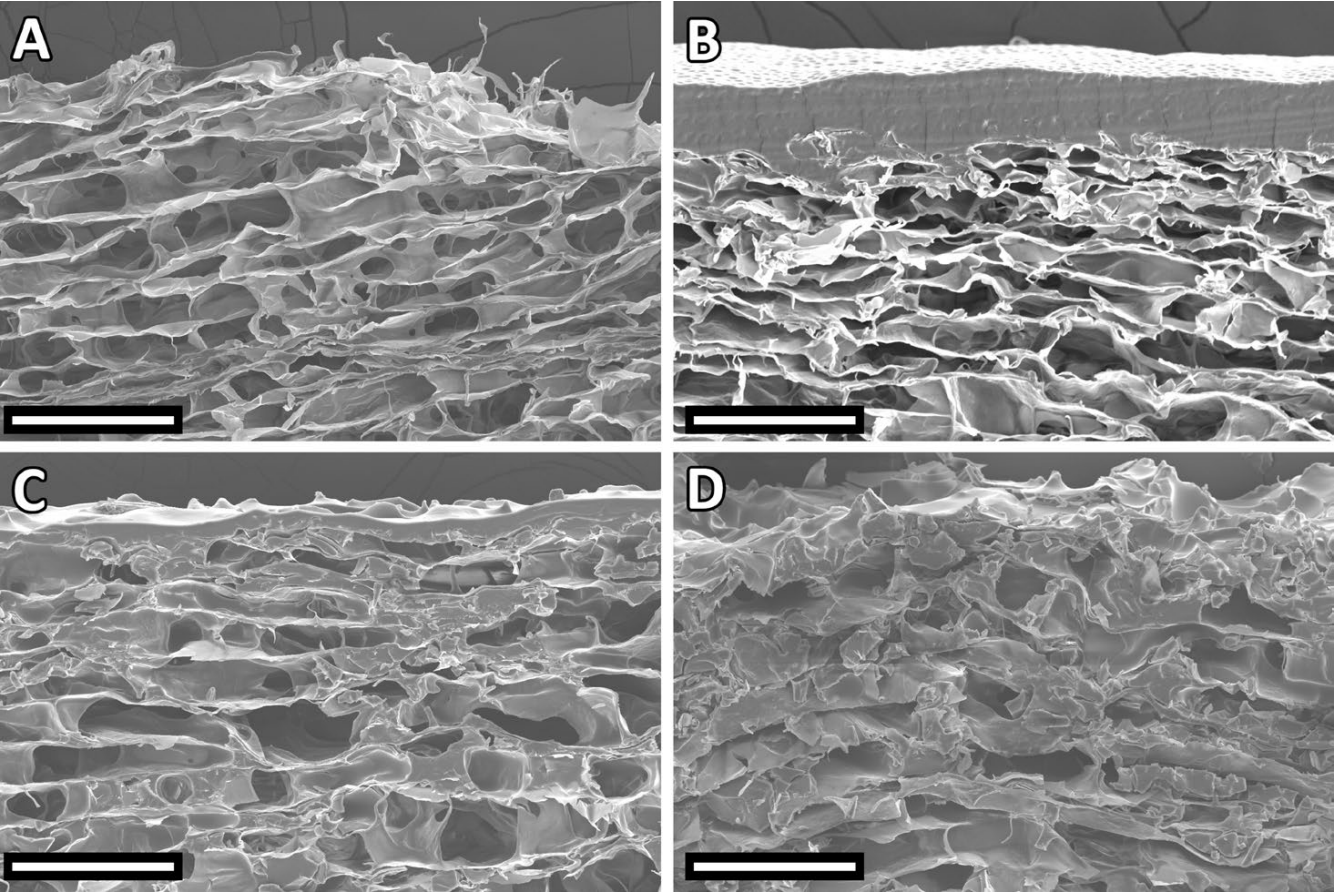
Illustrative SEM micrographs (mag. ×1000, bar 100 μm) of collagen membranes (A, B, C and D).

SEM images of cross‐sections of the prepared membranes after sterilization are shown in Figure 1. The average pore size determined from the SEM images indicated values of 40.2–60.4 μm (interquartile range; median 47.7 μm). These values may be influenced by the measurement method itself, as they are evaluated from 2D images and do not offer the possibility of evaluating the overall 3D structure. The results may also be affected by the orientation of the section and may be biased by artifacts arising from the cutting of the samples [26]. According to the measured pore size of the membranes, our experimental collagen membranes can be classified as microporous (pore size < 100 μm), which makes them more resistant to compression stress, more favorable to cell adherence due to the increased surface area, and prolongs their degradation rate. On the other hand, this architecture is prone to reduced vascularization and lower cell proliferation and activity [9, 27, 28].

Although the membranes were impregnated with the same amount of identically concentrated copolymers, the resulting layer thicknesses varied. The average thickness of each type of superficial layer was B: 45.7 (44.5–50.0) μm (median, interquartile range), C: 12.9 (11.9–14.4) μm, D: 4.2 (3.6–4.8) μm. The differences in the resulting layer thicknesses were probably due to the different viscosities of the copolymers used. The inherent viscosity of chloroform reported by the manufacturer for the l‐lactide/caprolactone copolymer (used in *membrane B*) is approximately 5–11 times higher than the viscosity of the d,l‐lactide/glycolide copolymers used for the preparation of membranes of type C and D. Therefore, the lower viscosity copolymer solutions may have penetrated the surface of the collagen sponge more. This phenomenon is most evident in the type D membrane, where the resulting thin layer on the surface was difficult to detect.

### 3.2 MRI Analysis—Degradation Profile of the Carp Collagen Experimental Membranes

**FIGURE 2:**
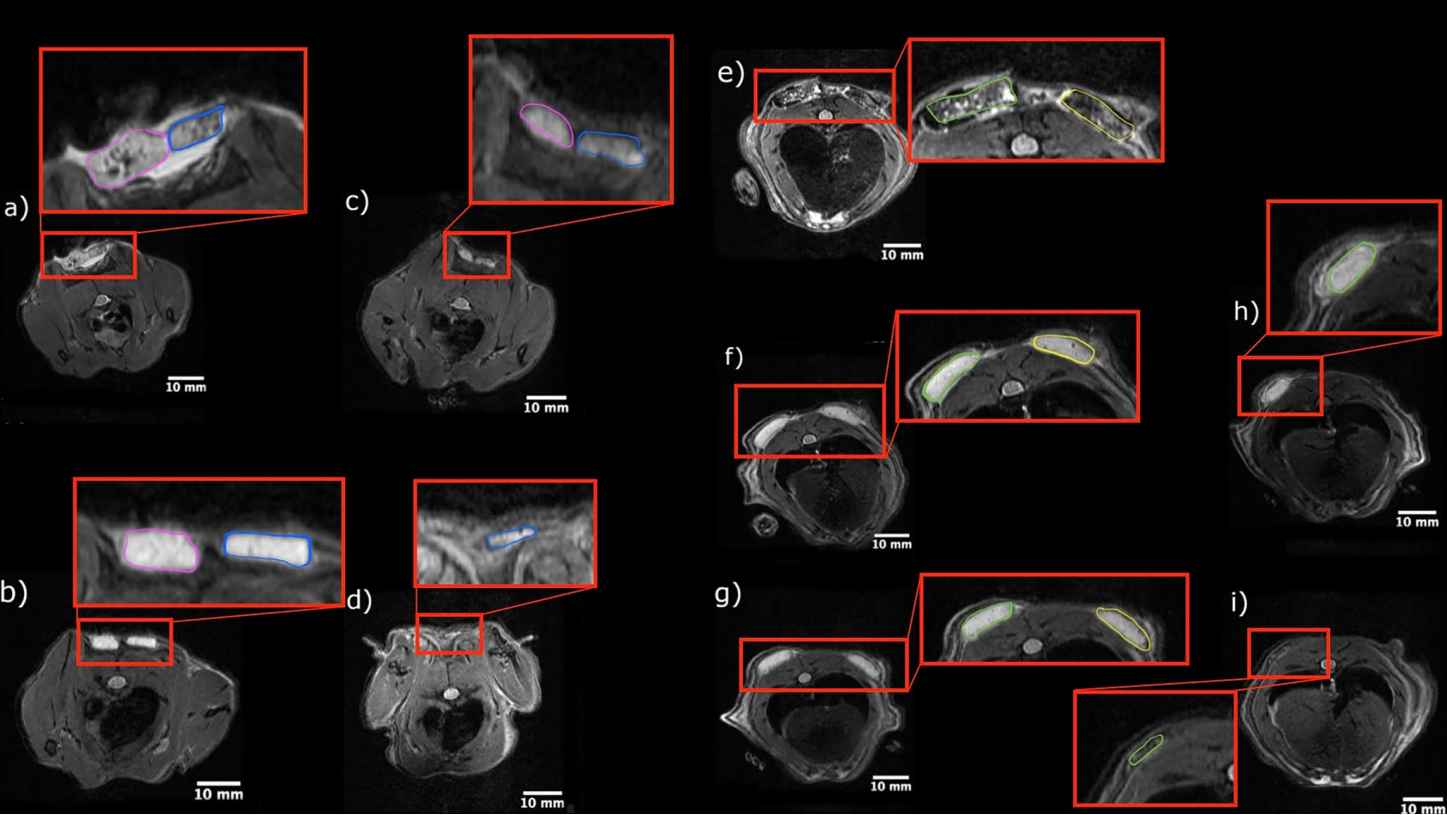
Representative MRI images (T2 sequence) of implanted collagen membranes. The visualized regions were zoomed in for clarity, and membranes are delineated with colored outlines corresponding to each membrane type: membrane A (violet), membrane B (blue), membrane C (green), and membrane D (yellow). Images are shown for Day 1 (a, e), Day 7 (b, f), Day 14 (c, g), 1 month (d, h), and 2 months (i). Membrane A is no longer visible at day 28 (d), and membrane D is undetectable by 2 months (i).

Collagen membranes degradation was assessed in vivo using micro‐MRI to measure real volume changes over a 16‐week period (Figure 2). Scanning was performed on Days 1, 7, 14, and 28, with extended intervals thereafter (8, 12, and 16 weeks), as the membranes were mostly degraded by 28 days. The largest proportion of degradation of all membrane types occurred within the first 14 days.

**FIGURE 3:**
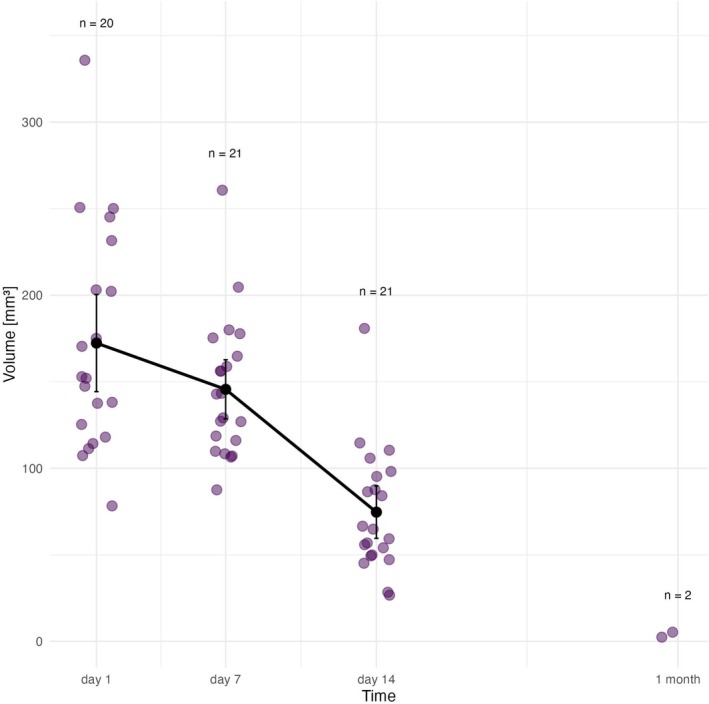
Degradation profile of membrane A, violet points represent individual observations (volume of membrane A in MRI) within each time point. Black vertical bars depict confidence intervals, black points EMM volume of membrane in particular time. At Day 1 area of membrane A implantation was not captured in rat no. 1.1. via MRI scan. Exact values are provided in the text. 1 month = 28 days.

*Membrane A* was detectable via micro‐MRI for up to 1 month; after which no discernible membranes were observed. Figure 3 and Table 1 illustrate the changes in membrane A volume, showing reduction over time.

**TABLE 1:** Pairwise post hoc comparisons of volume measurements for membrane a across time points.

Contrast	Estimate	SE	df	*t* Ratio	*p*	Lower 95% CI	Upper 95% CI
Days 1–7	28.4	16.6	57	1.72	0.207	−4.66	61.5
Days 1–14	98.2	16.1	57	6.09	< 0.001***	65.93	130.4
Days 7–14	69.7	11.8	57	5.88	< 0.001***	46.02	93.4

*Note:* Estimated differences in volume between time points, along with their standard errors (SE), degrees of freedom (df), *t* ratios (calculated as estimate/SE), *p*‐values, and 95% confidence intervals. Confidence intervals that do not include 0 support statistical significance.

*** Statistically significant differences (*p* < 0.001).

The EMM volume of membrane A was 172.3 mm^3^ (95% CI: 144.3–200.4 mm^3^). By Day 7, the EMM volume decreased to 143.9 mm^3^ (95% CI: 126.3–161.5 mm^3^), representing a 16.5% reduction from the original volume. However, in 4 rats (1.5, 2.1, 2.4, and 3.5), the membranes appeared larger on Day 7 than on Day 1, likely due to additional in vivo hydration, which caused membrane swelling. On Day 14, the EMM volume declined to 74.2 mm^3^ (95% CI: 58.3–90.1mm^3^), meaning a 56.9% reduction from the initial volume, and by Day 28, the membrane was almost completely degraded, detectable in only two rats (2.4 and 5.3 mm^3^; 97.8% reduction from the initial).

**FIGURE 4:**
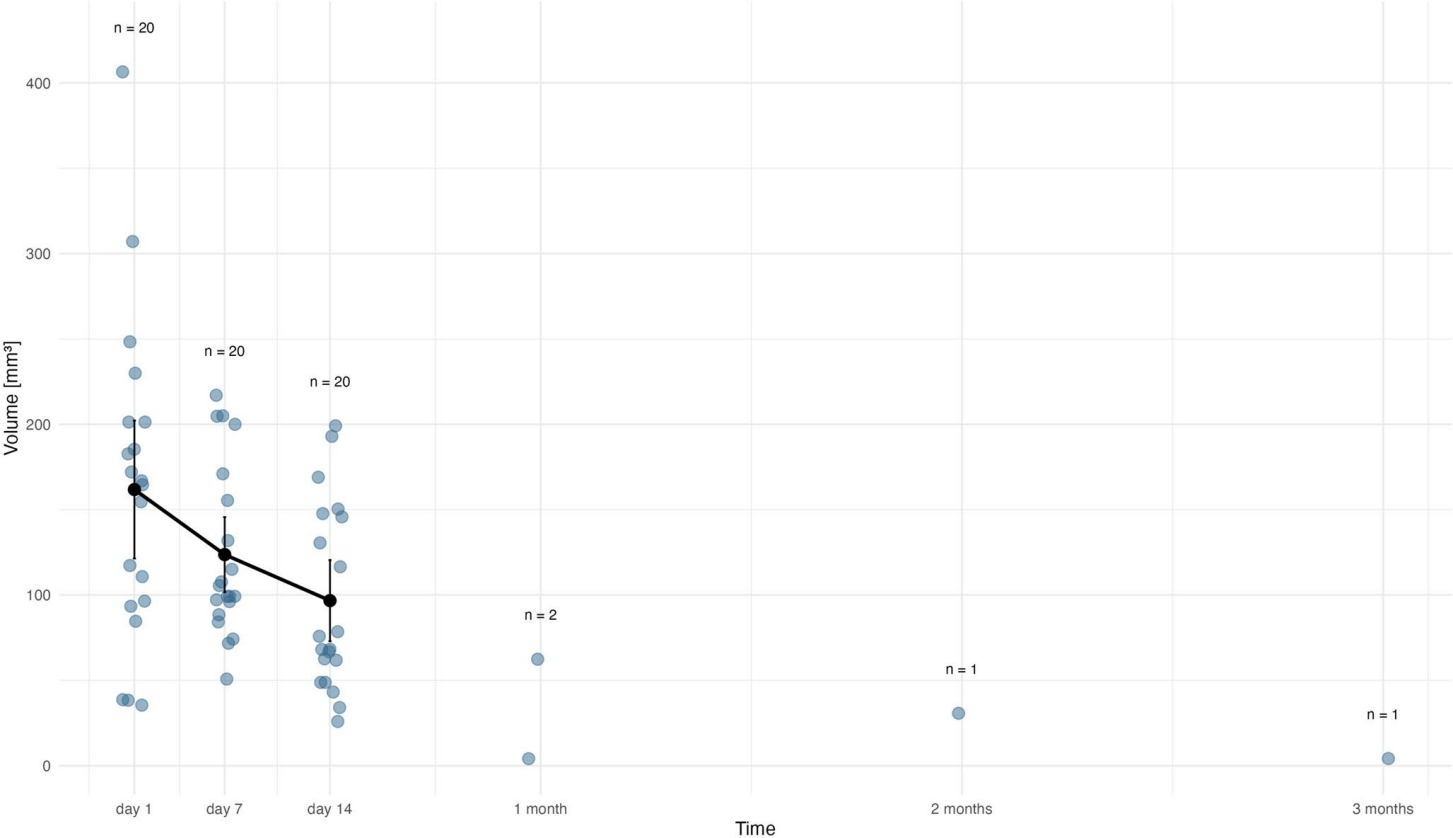
Degradation profile of membrane B, blue points represent individual observations (volume of membrane B in MRI) within each time point. Black vertical bars depict confidence intervals, black points EMM volume of membrane in particular time. At Day 1, the area of membrane B implantation was not captured in rat no. 1.1. via MRI scan. Exact values are provided in the text. 1 month = 28 days, 2 months = 54 days, 3 months = 84 days.

*Membrane's B* (Figure 4, Table 2) EMM volume on Day 1 was 161 mm^3^ (95% CI: 121.0–202.0 mm^3^). By Day 7, it decreased to 123.7 mm^3^ (95% CI: 102.0–146.0 mm^3^). Five observations (rats 1.3, 1.5, 2.1, 3.5, and 3.7) showed a larger membrane B volume on Day 7 compared to Day 1. On Day 14, the EMM volume decreased to 96.7 mm^3^ (95% CI: 73.0–120.0 mm^3^). That represents a 39.9% reduction from the initial size. One month after postimplantation, membrane B was detectable in two rats only, with volumes of 4 mm^3^ (rat 1.3) and 62 mm^3^ (rat 2.4). The membrane persisted in one rat (2.4) up to 3 months (4 mm^3^, 97.6% reduction from initial size).

**TABLE 2:** Pairwise post hoc comparisons of volume measurements for membrane B across time points.

Contrast	Estimate	SE	df	*t* Ratio	*p*	Lower 95% CI	Upper 95% CI
Days 1–7	38.1	23.0	57	1.66	0.230	−7.87	84.1
Days 1–14	65.1	23.4	57	2.78	0.020*	18.19	111.9
Days 7–14	26.9	16.2	57	1.67	0.227	−5.40	59.3

*Note:* Estimated differences in volume between time points, along with their standard errors (SE), degrees of freedom (df), *t* ratios (calculated as estimate/SE), *p*‐values, and 95% confidence intervals. Confidence intervals that do not include 0 support statistical significance.

* Statistically significant differences (*p* < 0.05).

**FIGURE 5:**
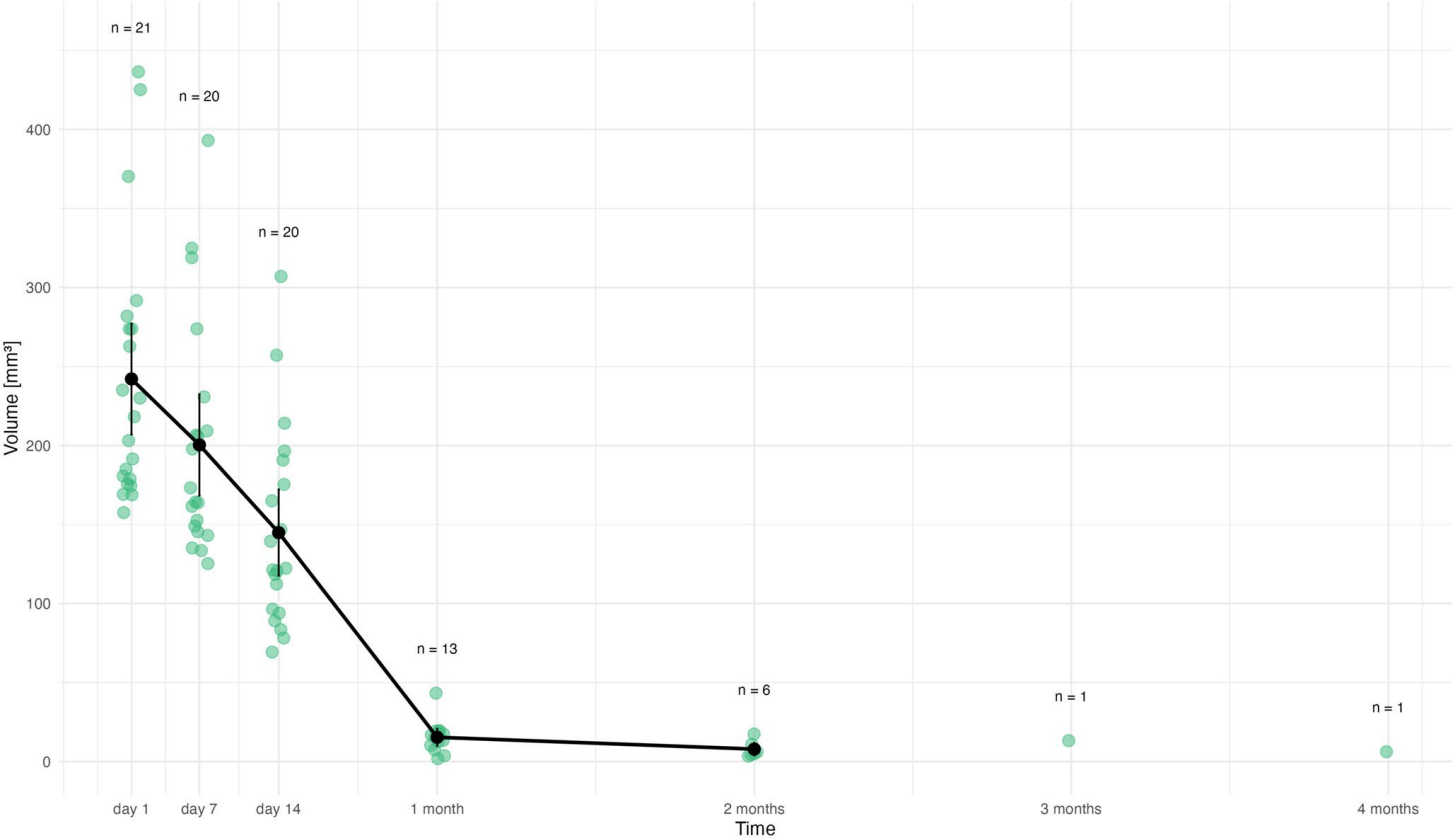
Degradation profile of membrane C, green points represent individual observations (volume of membrane C in MRI) within each time point. Black vertical bars depict confidence intervals, black points EMM volume of membrane in particular time. Exact values are provided in the text. 1 month = 28 days, 2 months = 54 days, 3 months = 84 days, 4 months = 112 days.

Figure 5 and Table 3 represent the volume change of *membrane C* in time. The initial EMM volume of membrane C was 242.1 mm^3^ (95% CI: 207.0–277.2 mm^3^). By Day 7, the volume decreased to 200.4 mm^3^ (95% CI: 168.4–232.4 mm^3^), which is 17.2% reduction. Unlike membranes A and B, none of the implanted membrane C showed a volume increase in the first week. On Day 14, EMM was 144.9 mm^3^ (95% CI: 117.6–172.2 mm^3^) representing 40.1% reduction from the initial volume. After 4 weeks (28 days), the scaffold was detectable in 16 cases (EMM = 15.3 mm^3^; 95% CI: 9.9–20.8 mm^3^), which equals to a 93.7% reduction. After 2 months (8 weeks), the membrane persisted in seven cases with EMM volume of 7.9 mm^3^ (95% CI: 3.9–11.8 mm^3^), that is, 96.6% smaller than the initial volume. One rat (1.5) retained a detectable membrane throughout the experiment, with volumes of 13 mm^3^ at 3 months (12 weeks) and 6 mm^3^ at 4 months (16 weeks).

**TABLE 3:** Pairwise post hoc comparisons of volume measurements for membrane C across time points.

Contrast	Estimate	SE	df	*t* Ratio	*p*	Lower 95% CI	Upper 95% CI
Days 1–7	41.72	23.87	75	1.75	0.412	−5.78	89.2
Days 1–14	97.23	22.33	75	4.35	< 0.001***	52.79	141.7
Days 1–28	226.77	17.83	75	12.72	< 0.001***	191.28	262.3
Days 1–56	234.25	17.74	75	13.21	< 0.001***	198.95	269.5
Days 7–14	55.52	21.15	75	2.62	0.076	13.43	97.6
Days 7–28	185.05	16.33	75	11.33	< 0.001***	152.56	217.6
Days 7–56	192.53	16.22	75	11.87	< 0.001***	160.25	224.8
Days 14–28	129.54	13.99	75	9.26	< 0.001***	101.71	157.4
Days 14–56	137.01	13.86	75	9.89	< 0.001***	109.43	164.6
Days 28–56	7.48	3.38	75	2.21	0.186	0.76	14.2

*Note:* Estimated differences in volume between time points, along with their standard errors (SE), degrees of freedom (df), *t* ratios (calculated as estimate/SE), *p*‐values, and 95% confidence intervals. Confidence intervals that do not include 0 support statistical significance.

*** Statistically significant differences (*p* < 0.001).

**FIGURE 6:**
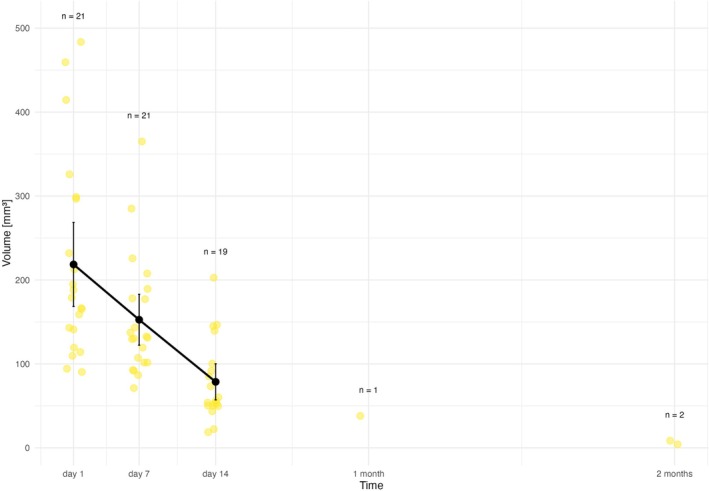
Degradation profile of membrane D, yellow points represent individual observations (volume of membrane D in MRI) within each time point. Black vertical bars depict confidence intervals, black points EMM volume of membrane in particular time. Exact values are provided in the text. 1 month = 28 days, 2 months = 54 days.

The initial EMM volume of *membrane D* 218.5 mm^3^ (95% CI: 168.4–269 mm^3)^ decreased after 7 days to 152.6 mm^3^ (95% CI: 122.3–183 mm^3^), that is, 30.1% volume reduction. In three rats (1.5, 2.2., 3.5) the volume on Day 7 was greater than on Day 1. The EMM volume on Day 14 was 78.9 mm^3^ (95% CI: 57.1–100 mm^3^), that is, 64.0% reduction from the initial size. After 1 month (4 weeks) membrane D was detected only in one animal (rat 1.1.) with a volume of 38 mm^3^ (82.6% reduction). However, after 2 months, the membrane was detected in two animals (rat 2.2., and 2.3.) with volumes of 4.18 and 8.46 mm^3^, respectively. The degradation profile is depicted in Figure 6 and Table 4.

**TABLE 4:** Pairwise post hoc comparisons of volume measurements for membrane D across time points.

Contrast	Estimate	SE	df	*t* Ratio	*p*	Lower 95% CI	Upper 95% CI
Days 1–7	65.9	29.4	58	2.24	0.073	7.04	125
Days 1–14	139.8	27.4	58	5.11	< 0.001***	85.07	195
Days 7–14	74.0	18.7	58	3.96	< 0.001***	36.60	111

*Note:* Estimated differences in volume between time points, along with their standard errors (SE), degrees of freedom (df), *t* ratios (calculated as estimate/SE), *p*‐values, and 95% confidence intervals. Confidence intervals that do not include 0 support statistical significance.

*** Statistically significant differences (*p* < 0.001).

Significant differences in membrane volumes were observed between membrane types at early time points. On Day 1, significant differences were noted between membranes A and C (*p* = 0.014), and membranes B and C (*p* = 0.019) despite the same initial “dry state” dimension of the implanted membranes. This observation is most probably caused by increased water absorption secondary to polymer coating of membrane C (d,l‐lactide‐glycolide 50:50). The second largest initial volumes had membrane D with the same types of polymers but in a different molar ratio (75:25). Glycolide is more hydrophilic than lactic acid and absorbs more water [29]. This can explain the greatest “hydrated” volume of membrane C, which has the greatest content of glycolide among all membrane types. After 7 days, the difference between membranes A and C persisted (*p* = 0.015), while the difference between membranes B and C became even more pronounced (*p* = 0.001). By Day 14, the membrane C volume was significantly different from that of membrane A (*p* < 0.001), B (*p* = 0.047), and D (*p* = 0.002), with 95% CIs well separated from zero (A–C: −102.2 to 39.1, B–C: −84.3 to −12.0, and C–D: 31.5–101.0). By 4 weeks postimplantation, the majority of the collagen membranes A, B, C, and D had undergone substantial resorption, resulting in an insufficient number of detectable remnants on MRI for robust statistical analysis. Detailed statistical results, displaying post hoc analysis including *p*‐values and CIs are accessible in Tables [Link], [Link].

Given the properties and half‐lives of the polymers used, we expected membrane B to have the longest duration and the slowest degradation rate. This was true for the first 14 days of observation, where the EMM volume of membrane B decreased by 34.9%, which was the lowest of all membranes tested (compared to A: 56.9%, C: 59.9%, D: 64%). However, after 1 month (4 weeks), the situation changed as membranes A and B were observed in only 2 cases, membrane D in 1 case, while membrane C was present in 13 of 21 cases. Another expectation was that membrane D (d,l‐lactide/glycolide in molar ratio 75:25) should degrade more slowly than membrane C (d,l‐lactide/glycolide in molar ratio 50:50), as observed by Rönkko et al. [30] in the rat model using similar molar ratios. The faster degradation was supposed to be due to an increased content of fast degrading glycolic acid in membrane C [14]. Surprisingly, membrane C was preserved for the longest period of time among all types.

Based on the EMMs of MRI volume data, the approximate degradation half‐lives (calculation explained in Section 2) of the collagen membranes were calculated to provide a clearer comparison of their degradation dynamics. Membrane A had an estimated half‐life of 10–11 days; membranes B and C approximately 18–21 days; however, membrane C retained a higher residual volume at later time points, suggesting slightly greater stability. In contrast, membrane D showed the fastest degradation, with a half‐life of approximately 9–10 days.

If our hypothesis regarding polymer penetration into the collagen membrane (mentioned at the beginning of the Results and Discussion section) holds true, we would expect membrane D, with the thinnest surface polymer layer in SEM images, to have the deepest polymer infiltration and thus the highest internal polymer content (compared to membrane B and C). Conversely, membrane B, which had the thickest surface coating, may have experienced very limited polymer penetration. In vivo, the superficial polymer layer of membrane B could have detached within the first 2 weeks, exposing the underlying pure collagen scaffold, which would then degrade similarly to membrane A. This could explain the initially slower degradation rate of membrane B (due to the presence of high‐molecular‐weight polymers) resulting in a longer half‐life but followed by faster disintegration. The detached polymer coating would likely remain undetectable by micro‐MRI due to its limited resolution (0.234 mm in‐plane, 1 mm slice thickness). However, this “polymer penetration hypothesis” does not explain the unexpectedly faster degradation of membrane D compared to membrane C. Our initial hypothesis—based on polymer composition and theoretical degradation kinetics—predicted the following order: B > D > C (from slowest to fastest degradation). This was not observed. An alternative explanation based on presumed polymer penetration (D > C > B) also failed to align with the results. These discrepancies suggest that additional, unidentified factors—such as scaffold microarchitecture or in vivo interactions—may have significantly influenced degradation behavior.

**FIGURE 7:**
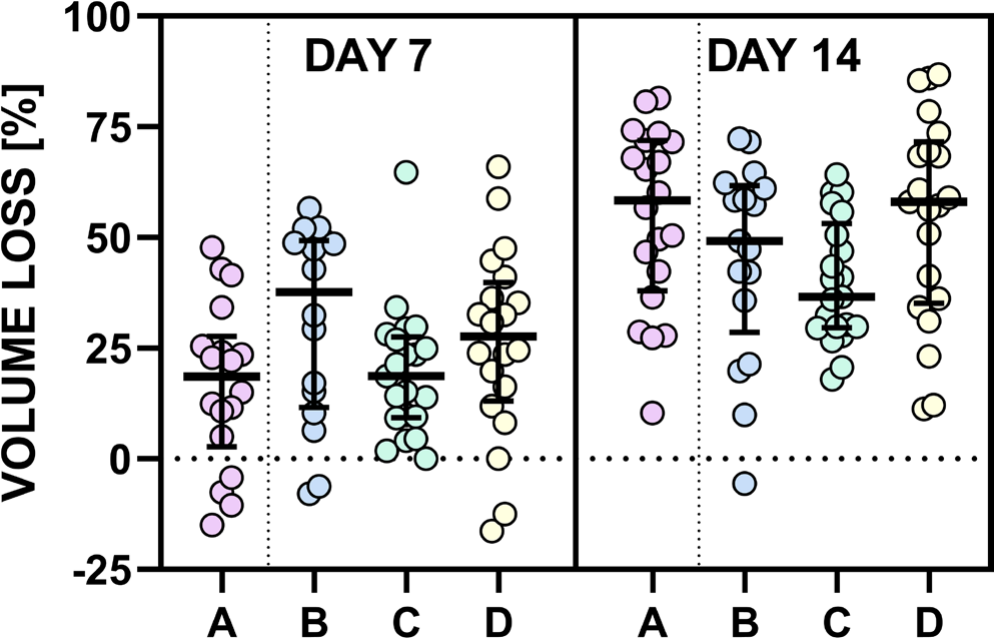
Relative volume loss of membranes at days 7 and 14 post‐implantation. Volume changes are expressed as relative loss compared to Day 1 for each individual sample. No statistically significant differences were observed between membrane types at either time point. The non‐parametric Kruskal–Wallis test was used, followed by Dunn's post hoc test with correction for multiple comparisons. Data are presented as median with interquartile range.

To investigate the unexpected persistence of Membrane C, volumetric data of all membrane types were evaluated at Day 1 postimplantation. An increase in membrane volume from the original approx. 96 mm^3^ was observed, with mean values (±SD) as follows: Membrane A: 172 ± 64 mm^3^, B: 162 ± 93 mm^3^, C: 242 ± 82 mm^3^, and D: 219 ± 118 mm^3^. These data indicate that membranes containing glycolide‐based copolymers (d,l‐lactide/glycolide 50:50 and 75:25) exhibited more pronounced swelling compared to collagen‐only or poly‐l‐lactide/PCL‐based membranes. The increased swelling is in accordance with the higher hydrophilicity and water absorption capacity of glycolide‐containing polymers [31, 32, 33]. To account for variability in initial swelling, relative volume changes were calculated for each membrane sample, normalized to Day 1 values. No statistically significant differences in relative volume change were found between membrane types on Days 7 and 14 postimplantation (see Figure 7). Please note, that longer time points could not be assessed using this approach due to limited data availability in most groups at 28 days (with the exception of membrane C). This suggests that the temporal progression of membrane volume is influenced by multiple factors beyond degradation alone, including the specific swelling characteristics of the polymer components. All membranes were prepared using the same method and polymer solution volume. Therefore, substantial differences in initial polymer distribution were not anticipated. As a result, techniques for direct compositional assessment (e.g., polymer quantification) were not included in the original study design. Based on current findings, further investigation using such methods is planned to verify the proposed explanation and to elucidate the relationship between swelling behavior and degradation kinetics in more detail.

Despite standardized membrane dimensions and hydration protocols, Day 1 MRI measurements exhibited notable intersample variability. This was likely influenced by individual differences in fluid uptake immediately postimplantation, small inconsistencies in membrane positioning, and technical limitations of 1 mm‐slice micro‐MRI, including partial volume effects. Additionally, volume estimates were based on manual segmentation in ImageJ, which may introduce subjective bias. While this baseline variability constrained our ability to detect subtle differences, overall degradation dynamics over time were consistent, and the superior persistence of membrane C was clearly observed.

Individual observed increases in volumes of membrane A, B, and D between Days 1 and 7 did not exceed 20 mm^3^ (i.e., 0.02 mL) in any observation. Statistical analysis revealed no significant differences between these two time points (Days 1 and 7) in any of the experimental membranes. Given the small magnitude of these volume increases and the possibility that not the whole membrane remnant could be captured, as well as the manual measurements of MRI images, the observed deviations are most likely attributable to measurement error rather than true changes in the volume of the membranes.

MRI observations suggest that the generally accepted minimal time frame of 4 weeks for critical initial bone healing may be insufficient for membranes A, B, and D in the context of GBR. However, the degradation dynamic may differ significantly when the membranes are placed on the bone surface. The bone‐adjacent layer of the membrane would be exposed to the “slower” bone cells and therefore this arrangement could lead to slower membrane degradation. That constitutes a key limitation of our study conducted on the subcutaneous model. Future studies should include implantation on bone defects (e.g., calvarial or mandibular) in larger animal models to assess the degree of osteogenesis and barrier function under clinical‐like conditions.

Another significant limitation that needs to be considered is the relatively small sample size per time point (7 animals per time point), which has an impact on the statistical power of our analyses, especially at the later intervals (i.e., after 4 weeks), where membrane disintegration reduced data availability. Additionally, this limitation likely contributed to the large variability observed within each group (Figures 3, 4, 5, 6), explained earlier in this section. Nevertheless, this study was designed as an initial pilot experiment to evaluate a novel carp collagen‐based membrane and assess, for the first time, its degradation in vivo using micro‐MRI as a noninvasive monitoring tool. The ethical constraints of animal use, combined with budgetary restrictions, limited our ability to expand group sizes. Future studies in an authentic osseous environment with larger cohorts should be performed to allow more robust, statistically supported conclusions.

It is also inevitable to remember that the degradation rate measured by MRI reflects the volumetric loss of the entire membrane, regardless of whether the degradation takes place internally or via superficial erosion.

### 3.3 MRI Analysis—T1 and T2 Signal Intensity Changes Over Time in Experimental Membranes

In addition to volume measurements, T1 and T2 intensities, as well as their standard deviations, were analyzed to determine whether any structural changes within the collagen scaffolds could be detected by this method. At the beginning of an experiment, a relatively low T1 signal (long T1 relaxation time) is expected, as there is a large number of crosslinks within the membrane and the collagen structure is relatively dense. Over time, T1 intensity is expected to increase as the collagen membrane loses its cross‐links [34]. T2 intensity is expected to increase with the loss of cross‐links due to fewer collagen‐water interactions and a higher proportion of freely mobile water molecules, which leads to a decrease in T2 relaxation time [35].

**FIGURE 8:**
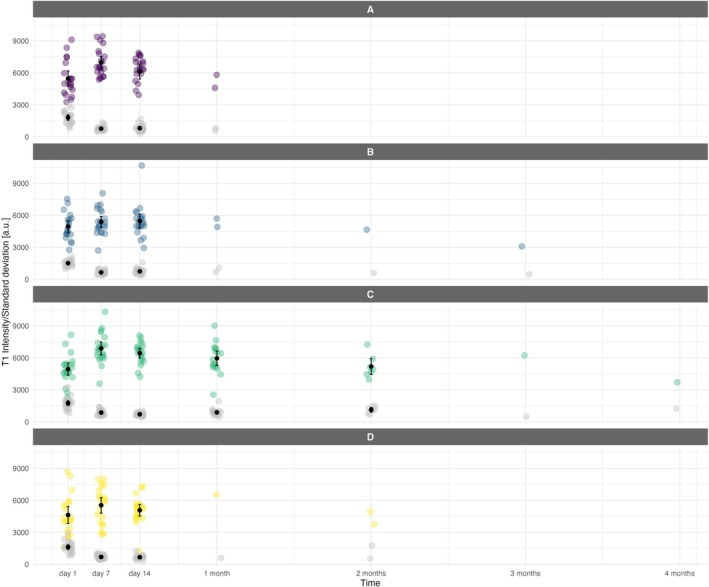
Change of T1 intensity and its standard deviation over time for each membrane type. Individual intensity observations are shown as colored points (violet = membrane A, blue = membrane B, green = membrane C, yellow = membrane D), while grey points represent the corresponding standard deviation values. Black points indicate the EMMs with their 95% confidence intervals where available. For membrane C, additional T1 intensity statistically significant difference not described in the main text: Day 7 versus 2 months (*p* = 0.007). Time points: 1 month = 28 days, 2 months = 56 days, 3 months = 84 days, 4 months = 112 days.

**FIGURE 9:**
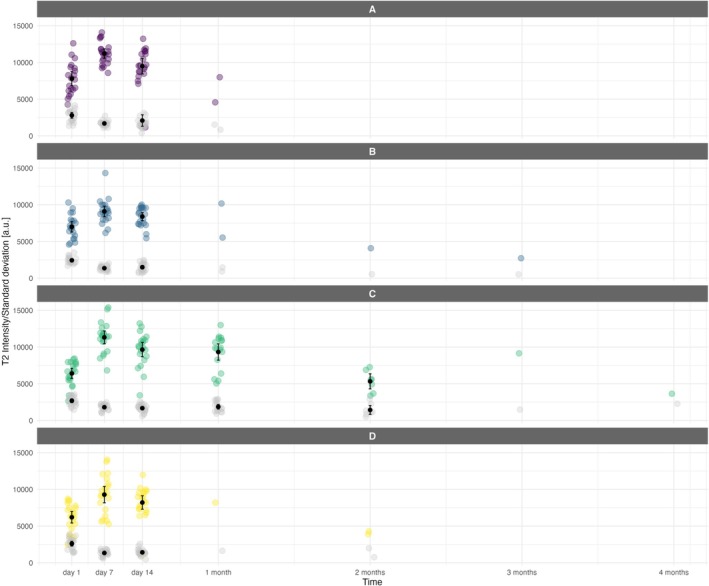
Change of T2 intensity and its standard deviation over time for each membrane type. Individual intensity observations are shown as colored points (violet = membrane A, blue = membrane B, green = membrane C, yellow = membrane D), while grey points represent the corresponding standard deviation values. Black points indicate the EMMs with their 95% confidence intervals where available. For membrane C, additional T2 intensity statistically significant differences not described in the main text include: Day 1 and 1 month, Day 7 and 2 months, Day 14 and 2 months, and between 1 month and 2 months (*p* < 0.001 for all comparisons). Time points: 1 month = 28 days, 2 months = 56 days, 3 months = 84 days, 4 months = 112 days.

Signal intensities in both T1‐ and T2‐weighted images increased in all membrane types 1 week postimplantation probably due to an initial degradation of collagen cross‐links and membrane swelling, that is, increased water content (Figures 8 and 9). This increase was statistically significant for membrane A (*p* = 0.004) and membrane C (*p* < 0.001) when measured in the T1 signal. T2 signal intensity values showed a highly significant difference between Days 1 and 7 in all membrane types (A, B, C, and D: *p* < 0.001). On Day 14, T2 intensities remained significantly higher than those on Day 1 in all membrane types (A: *p* = 0.05, B: *p* = 0.008, C: *p* < 0.001, D: *p* = 0.004). In contrast, significant changes in the T1 signal between Days 1 and 14 were observed only for membrane C (*p* < 0.001); no such differences were detected for membranes A, B, and D.

The signal increase in T1‐weighted images is somewhat controversial. T2‐weighted images confirmed increased content of water, which should cause rather hypointensity in T1‐weighted images. However, we do not know the true relaxation times of water entrapped in the collagen; moreover, the T1‐weighted sequence used had rather weak T1 weighting, as the sequence triggering did not allow for strong shortening of the repetition time. Therefore, the T1‐weighted sequence has rather a mixed T1/proton density weighting.

Due to the limited number of observations after 14 days for membranes A, B, and D, robust statistical analysis was possible only for membrane C (detailed in Figures 7 and 8). Even with fewer observations, the trend in the graphs shows a slight decrease in signal intensities over time, yet the values remain above baseline (Day 1) for both T1 and T2. This plateau is most evident for membrane C, which remained detectable on MRI for the longest period of time. These findings suggest that, after early structural disorganization, membrane C retains its residual structure for a more extended period compared to other membranes and reaches equilibrium, after which further structural breakdown occurs at a much slower rate. The subsequent decrease in signal is likely caused by complete membrane disintegration and therefore lack of recognition on MRI from surrounding soft tissue. This is in agreement with our MRI volume measurements, indicating that membranes A, B, and D are practically absent on MRI scans by 1 month. There were no statistically significant differences in T1 or T2 signal intensities between membrane types on Days 1 or 14. On Day 7, T1 and T2 signals of membranes A and C were significantly higher than those of membranes B and D. There were no significant differences between membranes A versus C and B versus D at that time point. There was not enough data to perform statistical and post hoc analysis for later time points.

While early trends in T1 and T2 intensities were observed (increase of the signals in T1‐ and T2‐weighted images in the first week), these parameters could not be reliably correlated with membranes degradation rates, or biocompatibility scores. The signal dynamics lacked consistency across membrane types. As such, T1/T2 intensity measurements, although measurable, provided no clear interpretative value in this pilot model and do not currently support any diagnostic or translational utility.

Standard deviations of T1 and T2 intensities were evaluated to assess scaffold homogeneity. All experimental membranes exhibited a decrease in standard deviation during the first week, after which the values stabilized. Day 1 values were significantly different (*p* < 0.001) from all other common time points evaluated (Days 7 and 14 in case of membranes A, B, C, and D), in both T1 and T2 intensities. For membrane C, the measurements on Day 1 also differed significantly from those on Day 28 (*p* = 0.001) and Day 56 (*p* = 0.002) for both T1 and T2. These results indicate homogenization of the implants within the first week, likely due to resorption of air bubbles and hematomas resulting from the surgical procedure.

### 3.4 Histological Analysis

Complementary histological analysis revealed generally low irritancy scores for all types of membranes. The tested GBR membranes were either non‐irritant or only slight‐irritant through the specific time points. The slight‐irritant scores of membranes A and B were on the lower limit (given scale for slight‐irritant 3–8.9), not exceeding 3.71. The irritancy scores of membranes C and D reached maximal values in the middle of the given range with EMM values of 6 and 4.56, respectively. Statistical analysis results of particular membrane types analyses are provided in the text below; post hoc analysis reflecting differences between the irritancy scores of the membranes at particular time points is provided in Tables [Link], [Link].

*Membrane A* showed the lowest irritancy score from all tested membranes at two time points: in Group 1 (4‐week observation period) and in Group 3 (16‐week observation period) with estimated means of 0.43 (95% CI: −0.14 to 1.00) and 0.72 (95% CI: −0.22 to 1.66), respectively. These results (close to 0) indicated its nonirritant properties, with the reaction of surrounding tissue equivalent to that of surgical incision with subcutaneous dissection (control). This corresponds to the high biocompatibility of the carp collagen sponge, as already indicated by the authors of the patent [12].

However, 12 weeks after implantation (Group 2), membrane A's EMM irritancy score was 3.71 (95% CI: 2.56–4.87), which is interpreted as a slight irritant (borderline with non‐irritancy). At this time point, membrane A had the highest irritancy potential of all tested GBR membranes. The variance of this group was not large, and there were no outliers. Six of seven rats in Group 2 had an irritancy score of membrane A between 3.0 and 6.0, which corresponds to a slightirritant potential (3–8.9) [22]. Therefore, Group 2 differed significantly from Group 1 (*p* < 0.001) and Group 3 (*p* < 0.001) in membrane A. This discrepancy is most likely due to sampling, identification variability, and the inherently subjective nature of the histological scoring system used in histological assessment. As the membranes were not labeled or stained prior to implantation, to eliminate possible degradation dynamic change, distinguishing partially resorbed membranes from surrounding granulation tissue was sometimes challenging. Furthermore, since all four membranes were implanted subcutaneously on the dorsal side of the rat, in relatively close proximity, it is also possible that the local inflammatory reaction to one membrane may have influenced adjacent sites.

*Membrane B* exhibited a trend toward a less irritant scaffold over time, as in the 4‐, 12‐, and 16‐week periods, it showed an EMM irritancy score of 3.71 (95% CI: 1.13–6.30), 2.71 (95% CI: 1.354.08), and 1.83 (95% CI: 0.40–3.26), respectively. However, there were no statistically significant changes between time points due to the high variance of the values. The irritancy potential switched from slight irritant in 4 weeks to nonirritant thereafter, which could implicate that the tissue reaction to membrane B diminishes over time as the membrane disintegrates. This pattern also supports the hypothesis proposed in the MRI section—that membrane B initially retained a superficial polymer layer, which may have detached within the first 2 weeks. The resulting mild irritant response observed at 4 weeks (EMM score 3.71) could reflect the presence of residual polymers or polymer degradation products. As the scaffold transitioned into a predominantly collagenous matrix (like membrane A), the tissue reaction diminished, resulting in a nonirritant profile at later time points.

At 4 weeks, the EMM for *membrane C* was 1.00 (95% CI: 0.17–1.83) and was therefore considered non‐irritant. Subsequently, the EMM increased to 3.14 (95% CI: 0.77–5.51) at 12 weeks and to 6.00 (95% CI: 4.16–7.84) at 16 weeks, and the membrane was labeled as a slight irritant at these two time points. The increase between Groups 1 and 3 (4–16 weeks) was considered highly statistically significant, with *p*‐value < 0.001. The CIs for the 12‐ and 16‐week observation points were relatively wide, resulting in no significant difference (*p* = 0.15). The degradation products of polymers contained in membrane C (poly‐d,l‐lactide and glycolide in a molar ratio 50:50) are d‐ or l‐lactic and glycolic acid, with both enzymatic and cellular (phagocytic cell induced) forms of degradation. These end substances are inherent to living organisms and can be processed by surrounding cells in the citrate cycle to carbon dioxide [36]. Taylor et al. [37] suggest a decrease in local pH (caused by these end products) as a possible cause of an increased inflammatory reaction. According to our histological sections, there was an increase in the number of macrophages and especially giant cells. In Group 1, there were no giant cells present in any rat, but in Group 3, giant cells were present in most of them. This is in concordance with the late‐onset foreign body tissue response. Up to date, there is no evidence that a low pH would itself trigger the formation of giant cells. On the other hand, we did not perform any method (e.g., FTIR—fourier transform infrared imaging) to determine the actual pH in tissues at a given time point to exclude causality of lower pH and increased inflammatory reaction. The observed increase in the irritancy score also cannot be explained by an increase of newly formed fibrous layer (which is included in the irritancy score and could possible increase it) in time because its thickness remained relatively the same from 4 weeks onward (Figure 13). This worsened biocompatibility of d,l‐lactide‐glycolide was already observed, but the mechanism is not clear [36].

The *membrane D* irritancy score showed variability over time with wide CIs, especially in the first two groups. EMM was 3.57 (95% CI: −0.22 to 7.36) at 4 weeks, then increased to 4.57 (95% CI: 0.87–8.27) at 12 weeks, giving a slight‐irritant status to membrane D. Subsequently, EMM decreased to 2.00 (95% CI: −0.26 to 4.26) suggesting a shift to non‐irritant response. However, due to high variability within groups, no statistically significant differences between time points were observed. Due to the higher content of d,l‐lactide and the lower glycolide content compared to membrane C (75:25 vs. 50:50), we expected a higher irritancy score for membrane D as in the study with a rat subconjunctival model where similar molar ratios were used [30]. Differences between membrane C and membrane D irritancy score were not statistically significant at 4 weeks, nor at 12 weeks. However, there was statistical significance at the 16 week time point (*p* = 0.031)—Table S6. We cannot explain this unexpected lower irritation profile of membrane D compared to membrane C. The probable explanation could be an insufficient sample size; therefore, high variance, and also (as in membrane A) the sampling and subjective nature of the histological scoring system used in assessment.

**FIGURE 10:**
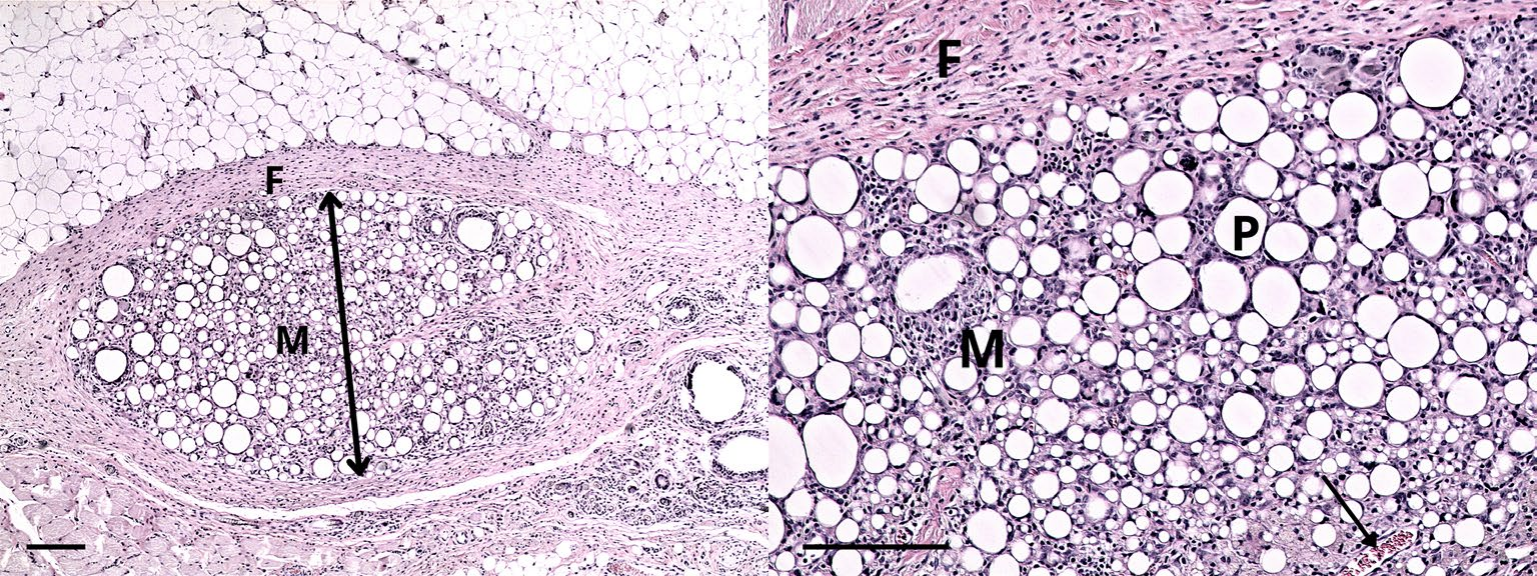
Representative histological sections of experimental carp collagen membrane D (impregnated with d,l‐lactide:glycolide in molar ratio 75:25) 4 weeks after implantation. Left: A ×40 magnification image showing the entire collagen membrane (M) surrounded by a fibrotic layer (F). Right: A ×100 magnification image highlighting detailed features of the collagen membrane, including visible pores (P), an ingrown vessel (indicated by an arrow), and the surrounding fibrotic layer (F). Polymeric layer is not visible. A 200 μm scale bar is shown in both images.

**FIGURE 11:**
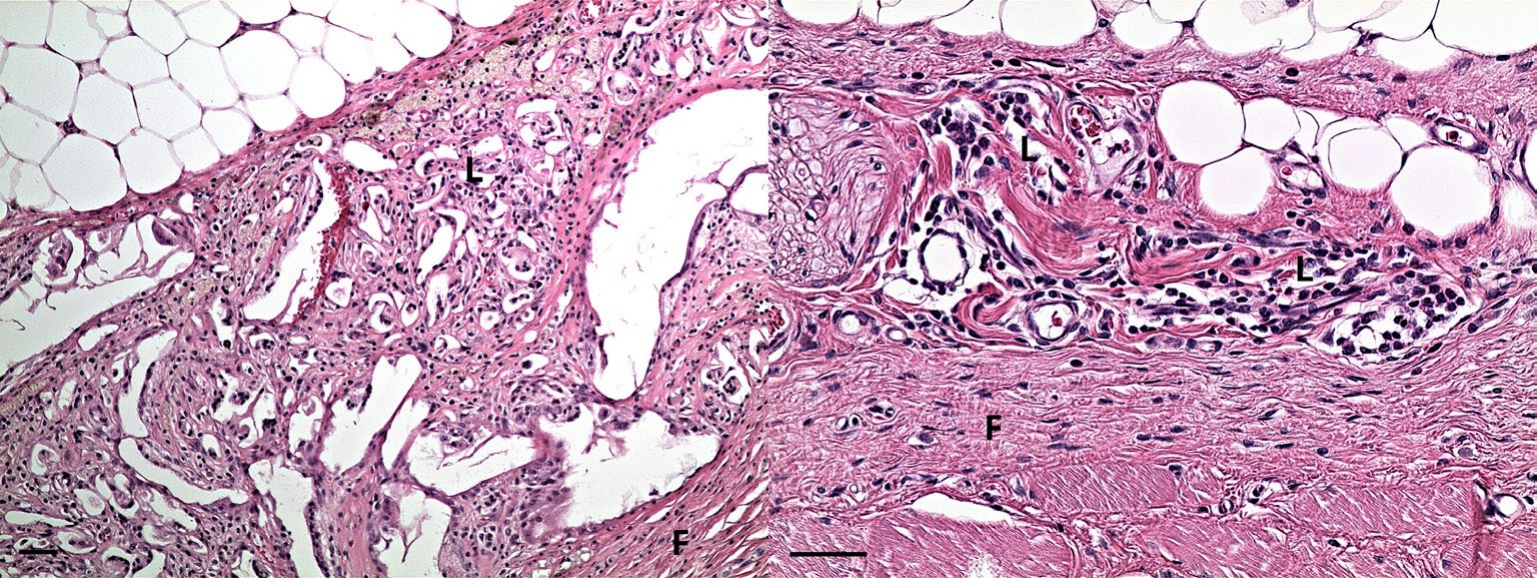
Representative histological section from the control site. Left: An overview of subcutaneous tissue after surgical preparation (magnification ×100). Right: A closer view of the same region (magnification ×200). Mild fibrosis (F) and scattered lymphocytic infiltration (L) are visible around the surgical site. Scale bars = 100 μm in both panels.

**FIGURE 12:**
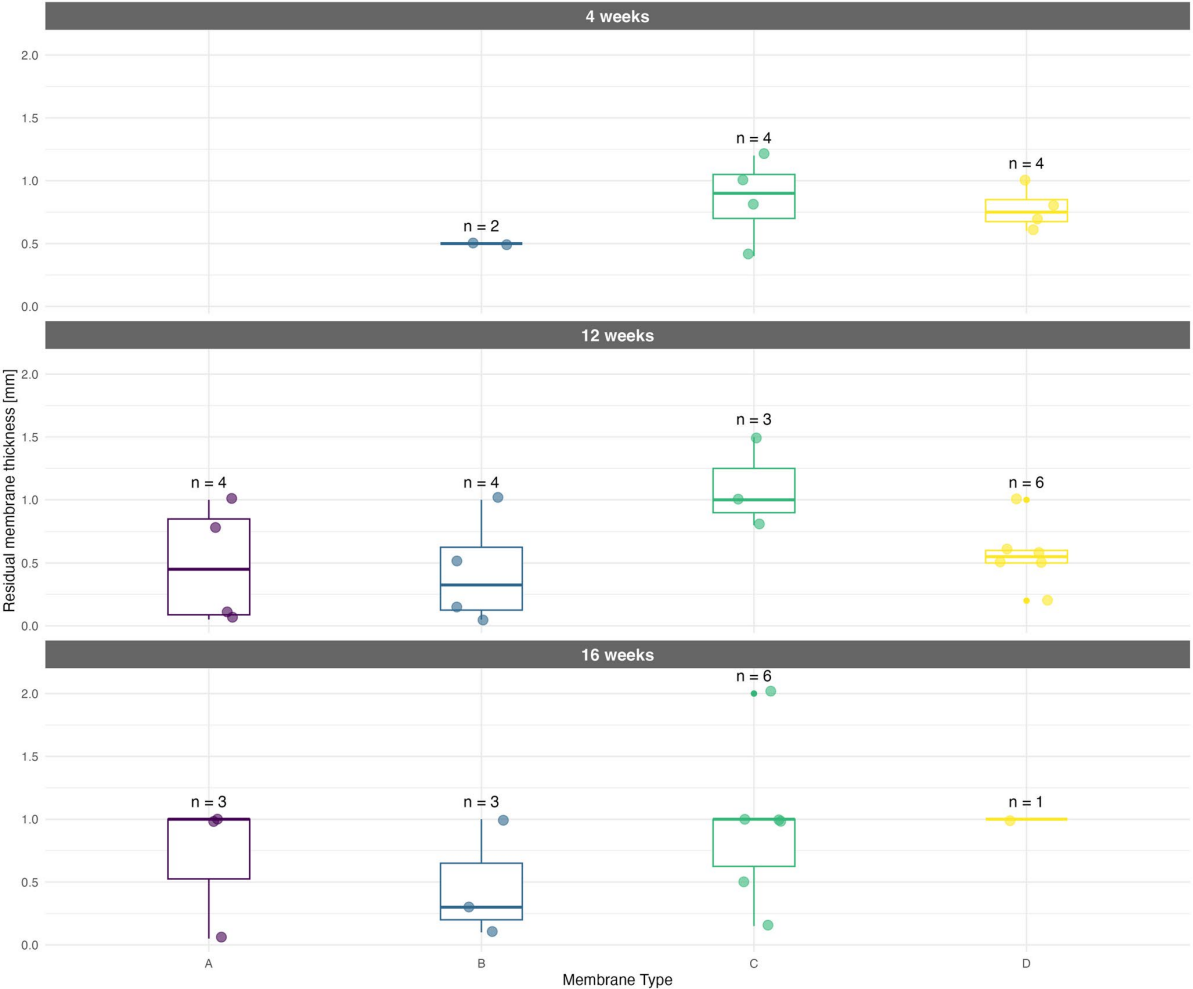
Residual membrane thickness at 4, 12, and 16 weeks postimplantation.

If the structure of particular collagen membranes was clearly discernible and nonfragmented in histological sections, the residual membrane thickness was measured (Figure 10). The control—skin incision and subcutaneous preparation without implanted material is—depicted in Figure 11. The summary results—including the number of observations, medians, and interquartile ranges at particular time points (4, 12, and 16 weeks) are displayed in Figure 12 (detailed numeric values are provided in Table S7). Although formal statistical analysis was not performed due to sparse data, trends are evident in the data visualization. Membrane A was undetectable after 4 weeks in all samples; however, at 12 weeks, three cases were observed with a median residual thickness of 0.1 mm. After 16 weeks, only one observation was recorded (median thickness 0.05 mm). The absence of membrane A at 4 weeks could reflect complete resorption; however, MRI captured a membrane in a rat of that group (rat 1.1). Membrane A reappearance at 12 weeks—where no remnants would be expected based on degradation kinetics—is consistent with the previously discussed histology analysis limitations in the section on irritancy scores. The lack of pre−/peri‐operative staining and the close proximity of implants may have contributed to sampling bias and misinterpretation of collagen membrane remnants. Membranes B and D showed a consistent decrease in residual thickness over time. Membrane C exhibited the greatest residual thickness across all time points, and its membrane was still microscopically clearly discernible in most rats even after 16 weeks. These results are in agreement with the micro‐MRI volume measurements, where membrane C maintained structural integrity longer than all other tested membrane types.

The higher number of membrane residues in histological sections compared to MRI images may be caused by the fact that the micro‐MRI slices were 1 mm thick; given the resolution of the scanner, the smaller residues of the membranes (i.e., the majority of them) could not be visualized.

Boxplots (with highlighted medians) show residual membrane thickness for four membrane types at each time point, with individual observations color‐coded (violet = membrane A, blue = membrane B, green = membrane C, yellow = membrane D). The number of observations (n) is displayed above each boxplot. Note that at 4 weeks, no observations were recorded for membrane A.

**FIGURE 13:**
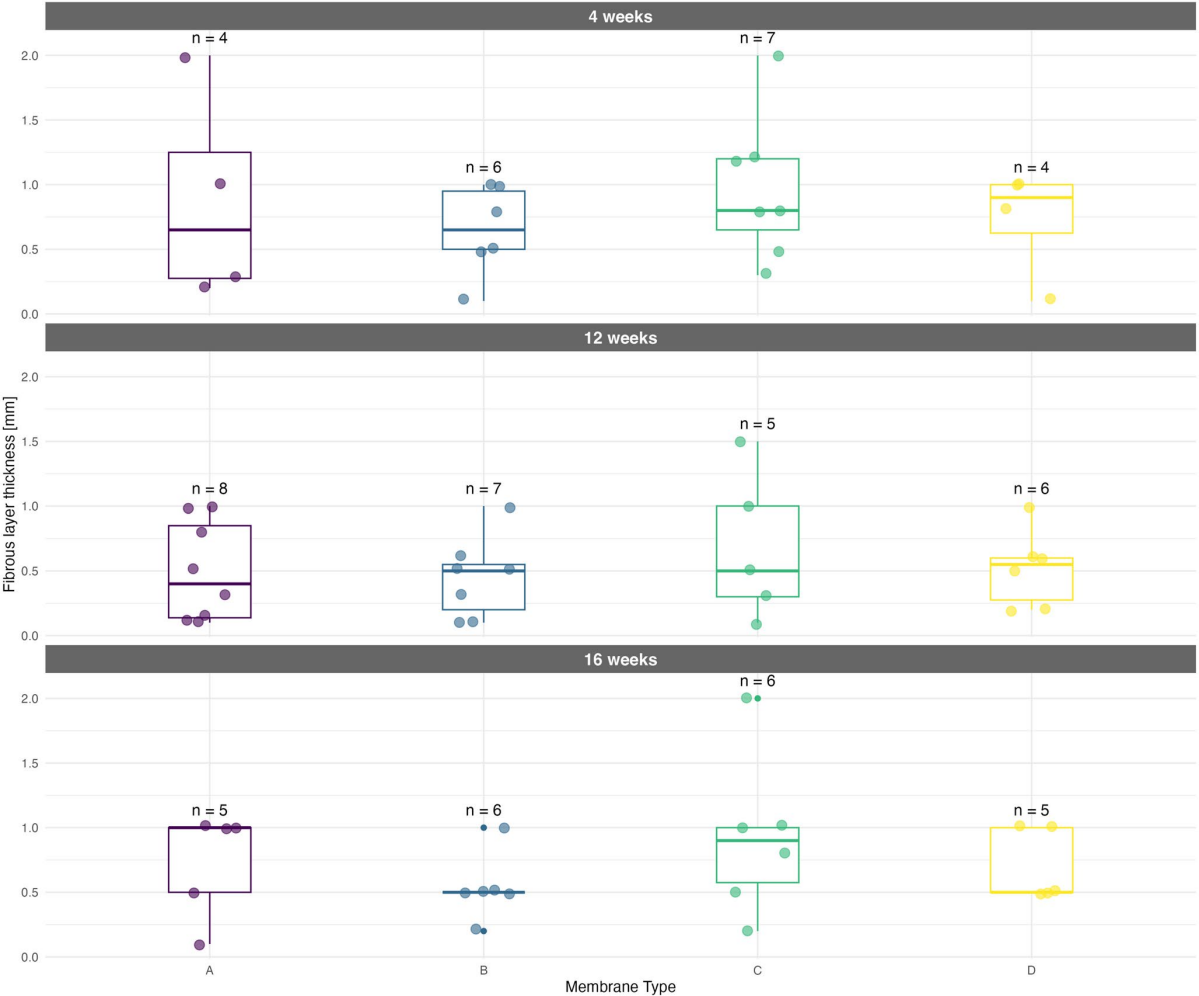
Fibrous layer thickness at 4, 12, and 16 weeks postimplantation.

As an additional parameter, the thickness of the newly formed fibrous layers around the implanted membranes was measured in histological sections. It was present in most of the samples. The statistical analysis did not result in any significant differences between membranes at particular time points, nor between time points within a particular membrane type. However, it is discernible from the median values that membrane C tends to induce the most extensive collateral fibrosis of all the variants tested (medians 0.9, 0.5, and 1.00 at 4, 12, and 16 weeks, respectively). These findings are also in concordance with the highest number of macrophages as well as giant cells in membrane C histological slices, which are responsible for foreign body reaction (FBR) and subsequent fibrous layer formation. Membrane A and membrane B yielded the lowest medians for the thickness of the fibrosis throughout the entire observation period, not exceeding 0.65 mm at any given time point. This limited tissue response is consistent with their lower irritancy scores compared to membranes C and D. The graphical interpretation of results is shown in Figure 13 and detailed numeric values (numbers of observation, medians, IQRs of membrane thickness an fibrous layer thickness) in Table S7.

The fibrous layer is crucial for the stability of the implanted membrane location; therefore, its proper function in GBR serves as well as a barrier to cytotoxic response from infiltrating immune cells. However, excessive FBR could impair the biocompatibility of the implant and even result in its failure [38]. Although the presence of fibrous encapsulation and foreign body giant cells indicates a moderate immune reaction, similar tissue responses have been reported in a rat model for resorbable bovine and porcine cross‐linked collagen membranes currently widely used in clinical practice [39], as well as marine collagen scaffolds [40].

With caution, we can interpret from the median values that the fibrous layer is relatively stable after 4 weeks and does not expand further over time. This claim is also supported by the lack of statistical significance between time points. Taken together with low irritancy scores for all the tested membranes (nonirritant or slight‐irritant) we can imply that in the case of these experimental membranes, the FBR was limited, not excessive, and did not affect the subjects (rats) negatively. Further studies with larger, more balanced sample sizes in a larger animal model are necessary to confirm these preliminary findings.

Boxplots (with highlighted medians) show the thickness of fibrosis around implanted scaffolds for four membrane types at each time point, with individual observations color‐coded (violet = membrane A, blue = membrane B, green = membrane C, yellow = membrane D). The number of observations (n) is displayed above each boxplot.

## 4 Conclusion

High demands are placed on collagen membranes, as the most commonly used products for TERM. As new products emerge, there is an increasing need for fast and effective monitoring of their degradation and biocompatibility assessment. Traditional histological evaluation requires a large number of experimental models in order to generate a sufficient number of histological slices over short time periods to follow product degradation. This is a demanding process both in terms of time and expenses. In our experimental study, where carp collagen membrane and its polymer (d,l‐lactide, l‐lactide, glycolide, and caprolactone in various molar ratios) impregnated variants were tested, we suggested micro‐MRI as an effective non‐invasive alternative for monitoring of membrane degradation in vivo, with no need for animal sacrifice, until the dimensions of remnants reach the resolution of the scanner. However, small collagen membrane fragments (< 1 mm) were identified only histologically, with no corresponding micro‐MRI signal. This suggests that micro‐MRI is effective in capturing overall degradation trends, but may underestimate the presence of residual material at late stages due to the partial volume effect. That said, the biological relevance of such submillimeter remnants is questionable: this pilot study used a small‐animal model (membranes area in dry state was 8 × 6 mm), while in clinical reality GBR membranes are much greater in size. At such scales, membrane fragments below 1 mm likely do not contribute meaningfully to barrier function or even structural support, which is most critical for GBR. In future studies involving larger animal models and clinically relevant membrane sizes, micro‐MRI may, therefore, provide a more proportionate and translationally valid readout of membrane persistence. Nevertheless, micro‐MRI does not allow assessment of biocompatibility and host tissue reaction, which are key domains where histology remains the gold standard.

Taken together, MRI and histological findings suggest that polymer modification did not uniformly prolong experimental GBR membrane persistence as expected: only membranes B and C demonstrated extended half‐lives, only membrane C retained substantial residual volume at later time points, indicating superior long‐term stability. This suggests that membrane C would most likely provide a more sustained barrier function during the critical early phase of bone regeneration. However, this benefit was accompanied by increased local irritancy, manifested as a delayed inflammatory response, and therefore reduced biocompatibility (classified as a slight‐irritant). In contrast, membrane B—despite its prolonged half‐life—did not demonstrate clear advantages over the control, membrane A, in terms of detectability on micro‐MRI from 4 weeks onward or tissue response. Membrane D, although polymer modified, demonstrated the shortest half‐life (~9–10 days) and no improvement in tissue response, behaving comparably to membrane A. These findings indicate that the effects of polymer addition are highly dependent on the polymer type and its interaction with the collagen membrane architecture.

All the observations were confined to subcutaneous tissue in an animal model with a relatively small sample size. Therefore, further experiments in an authentic bony environment with a larger sample size are needed to assess the bone healing capacity of these membranes. Despite these constraints, the observed degradation patterns and tissue responses provide valuable insight into the material's performance and feasibility as a GBR membrane.

## Disclosure

The collagen sponge evaluated in this study for its degradation rate in a rat model is protected under patent number CZ2019777A3. One of the authors (T.S.) of this article is one of the inventors of this patent. This potential conflict of interest has been disclosed in accordance with our institutional policies and relevant ethical guidelines. All efforts have been made to ensure the integrity and objectivity of the study, including independent data collection and analysis.

## Conflicts of Interest

One of the authors (T.S.) is a co‐inventor of a patent (CZ2019777A3) covering the collagen sponge from which the experimental membranes evaluated in this study were fabricated. All data collection and analysis were performed independently to ensure objectivity.

## Supporting information


**Table S1:** Pairwise post hoc comparisons of micro‐MRI volume measurements for membranes A, B, C, and D at Day 1 postimplantation. The table shows the estimated differences in volume between membrane types, along with their standard errors (SE), degrees of freedom (df), *t* ratios (calculated as estimate/SE), *p*‐values, and 95% confidence intervals. *Statistically significant differences (*p* < 0.05). Confidence intervals that do not include 0 support statistical significance.

**Table S2:** Pairwise post hoc comparisons of micro‐MRI volume measurements for membranes A, B, C, and D at Day 7 postimplantation. The table shows the estimated differences in volume between membrane types, along with their standard errors (SE), degrees of freedom (df), *t* ratios (calculated as estimate/SE), *p*‐values, and 95% confidence intervals. *Statistically significant differences (*p* < 0.05) and highly significant differences (***p < 0.001). Confidence intervals that do not include 0 support statistical significance.

**Table S3:** Pairwise post hoc comparisons of micro‐MRI volume measurements for membranes A, B, C, and D at Day 14 postimplantation. The table shows the estimated differences in volume between membrane types, along with their standard errors (SE), degrees of freedom (df), *t* ratios (calculated as estimate/SE), *p*‐values, and 95% confidence intervals. *Statistically significant differences (*p* < 0.05) and highly significant difference (***p < 0.001). Confidence intervals that do not include 0 support statistical significance.

**Table S4:** Pairwise post hoc comparisons of irritancy score for membranes A, B, C, and D at 4 weeks (1 month) postimplantation. The table shows the estimated differences in irritation score between membrane types, along with their standard errors (SE), degrees of freedom (df), *t* ratios (calculated as estimate/SE), *p*‐values, and 95% confidence intervals. *Statistically significant differences (*p* < 0.05) and highly significant difference (***p < 0.001). Confidence intervals that do not include 0 support statistical significance.

**Table S5:** Pairwise post hoc comparisons of irritancy score for membranes A, B, C, and D at 12 weeks (3 months) postimplantation. The table shows the estimated differences in irritation score between membrane types, along with their standard errors (SE), degrees of freedom (df), *t* ratios (calculated as estimate/SE), *p*‐values, and 95% confidence intervals. There are no statistically significant differences at this time point.

**Table S6:** Pairwise post hoc comparisons of irritancy score for membranes A, B, C, and D at 16 weeks (4 months) postimplantation. The table shows the estimated differences in irritation score between membrane types, along with their standard errors (SE), degrees of freedom (df), *t* ratios (calculated as estimate/SE), *p*‐values, and 95% confidence intervals. *Statistically significant differences (*p* < 0.05) and highly significant difference (***p < 0.001). Confidence intervals that do not include 0 support statistical significance.

**Table S7:** Summary of residual membrane and newly formed fibrous layer thickness. For each membrane type (A, B, C, and D), data are presented at three time points (4 weeks = 1 month, 12 weeks = 3 months, and 16 weeks = 4 months). For each time point, the table displays the median thickness (with interquartile range) and the number of observations for both the residual membrane and the surrounding fibrous layer.

## Data Availability

The data that support the findings of this study are available from the corresponding author upon reasonable request.
