# Supplementary figures and images for: In Vivo Degradation and Local Tissue Response of Experimental Carp Collagen Membranes: Micro‐MRI and Histological Analysis
Source: Biopolymers. 2025 Aug 18;116(5):e70045. doi: 10.1002/bip.70045 (PMC12359101; doi:10.1002/bip.70045)

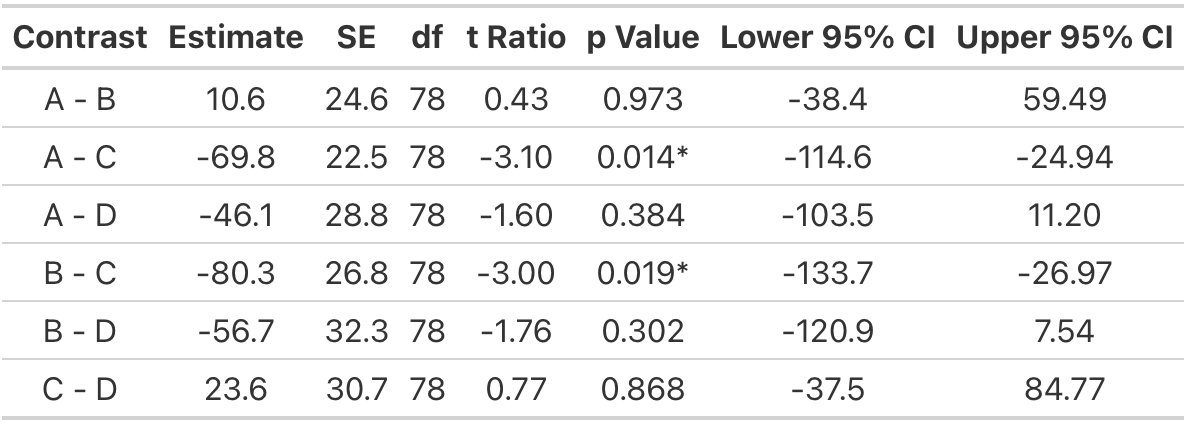

Supplement: Supplementary file 1 — Table S1: Pairwise post hoc comparisons of micro‐MRI volume measurements for membranes A, B, C, and D at Day 1 postimplantation. The table shows the estimated differences in volume between membrane types, along with their standard errors (SE), degrees of freedom (df), t ratios (calculated as estimate/SE), p‐values, and 95% confidence intervals. *Statistically significant differences (p < 0.05). Confidence intervals that do not include 0 support statistical significance. [file BIP-116-e70045-s006.png]

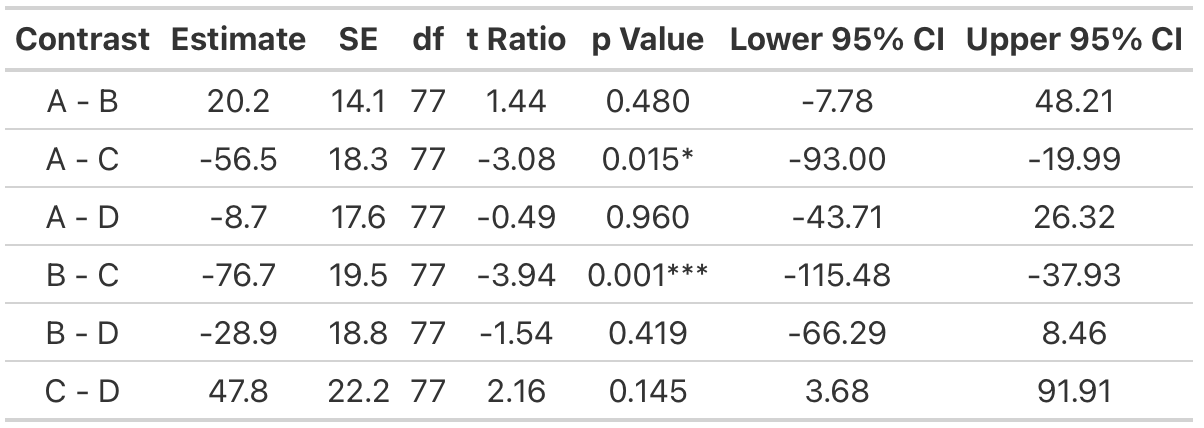

Supplement: Supplementary file 2 — Table S2: Pairwise post hoc comparisons of micro‐MRI volume measurements for membranes A, B, C, and D at Day 7 postimplantation. The table shows the estimated differences in volume between membrane types, along with their standard errors (SE), degrees of freedom (df), t ratios (calculated as estimate/SE), p‐values, and 95% confidence intervals. *Statistically significant differences (p < 0.05) and highly significant differences (***p < 0.001). Confidence intervals that do not include 0 support statistical significance. [file BIP-116-e70045-s001.png]

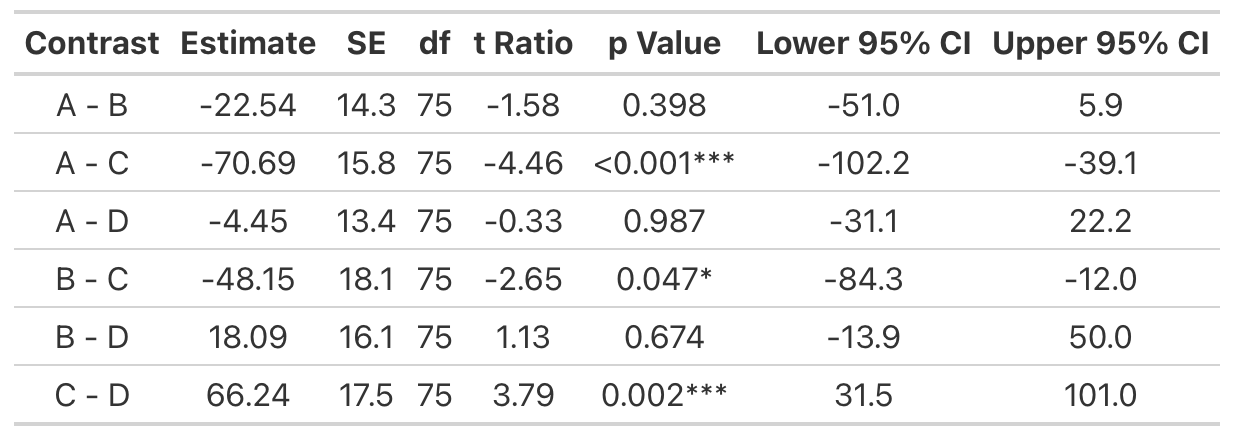

Supplement: Supplementary file 3 — Table S3: Pairwise post hoc comparisons of micro‐MRI volume measurements for membranes A, B, C, and D at Day 14 postimplantation. The table shows the estimated differences in volume between membrane types, along with their standard errors (SE), degrees of freedom (df), t ratios (calculated as estimate/SE), p‐values, and 95% confidence intervals. *Statistically significant differences (p < 0.05) and highly significant difference (***p < 0.001). Confidence intervals that do not include 0 support statistical significance. [file BIP-116-e70045-s007.png]

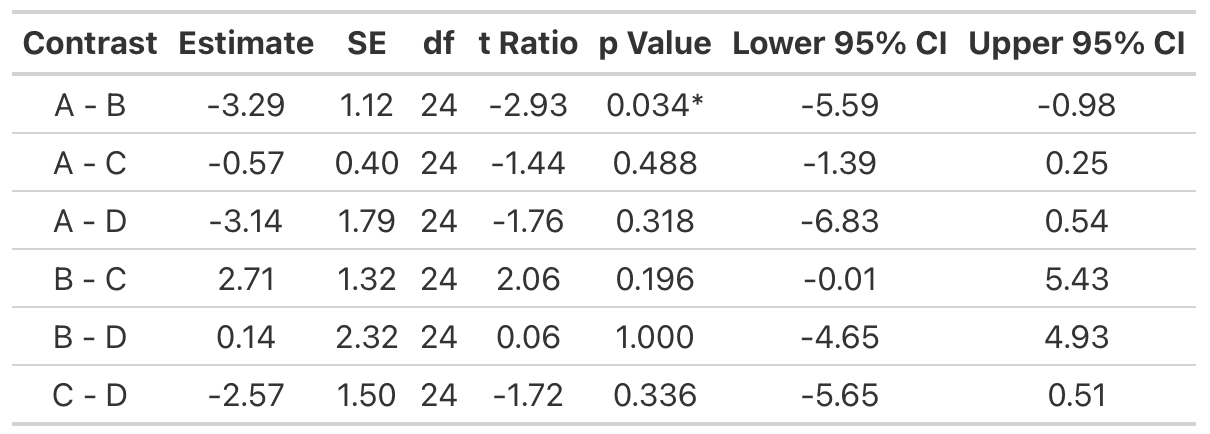

Supplement: Supplementary file 4 — Table S4: Pairwise post hoc comparisons of irritancy score for membranes A, B, C, and D at 4 weeks (1 month) postimplantation. The table shows the estimated differences in irritation score between membrane types, along with their standard errors (SE), degrees of freedom (df), t ratios (calculated as estimate/SE), p‐values, and 95% confidence intervals. *Statistically significant differences (p < 0.05) and highly significant difference (***p < 0.001). Confidence intervals that do not include 0 support statistical significance. [file BIP-116-e70045-s003.png]

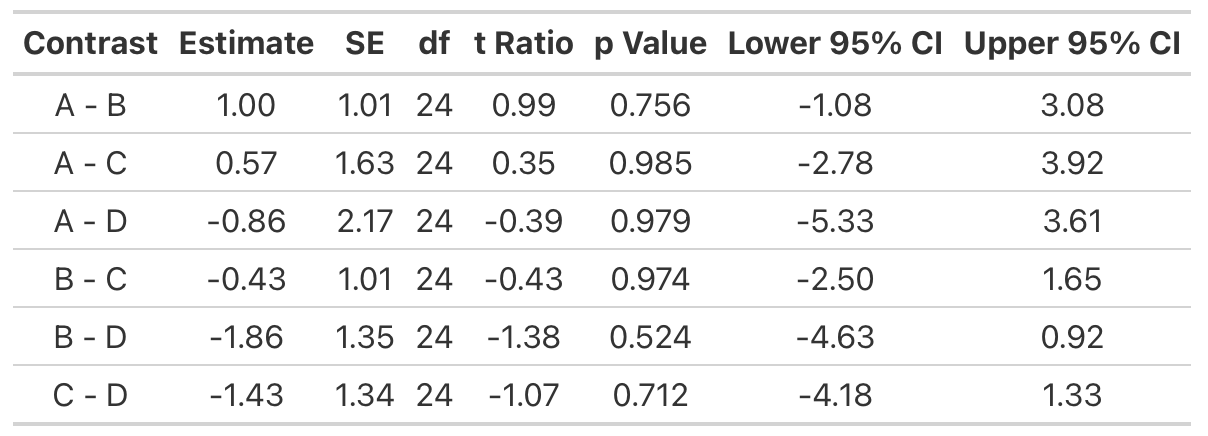

Supplement: Supplementary file 5 — Table S5: Pairwise post hoc comparisons of irritancy score for membranes A, B, C, and D at 12 weeks (3 months) postimplantation. The table shows the estimated differences in irritation score between membrane types, along with their standard errors (SE), degrees of freedom (df), t ratios (calculated as estimate/SE), p‐values, and 95% confidence intervals. There are no statistically significant differences at this time point. [file BIP-116-e70045-s005.png]

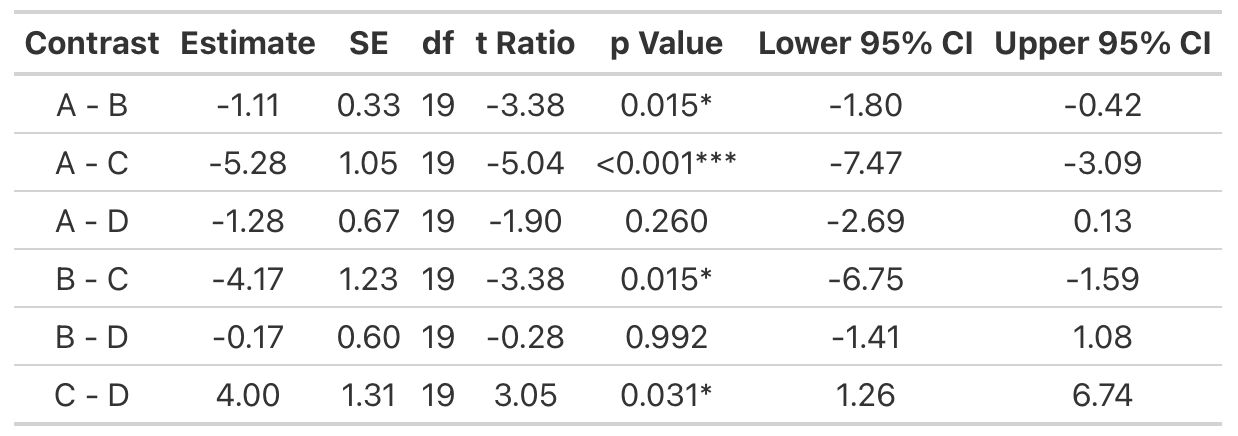

Supplement: Supplementary file 6 — Table S6: Pairwise post hoc comparisons of irritancy score for membranes A, B, C, and D at 16 weeks (4 months) postimplantation. The table shows the estimated differences in irritation score between membrane types, along with their standard errors (SE), degrees of freedom (df), t ratios (calculated as estimate/SE), p‐values, and 95% confidence intervals. *Statistically significant differences (p < 0.05) and highly significant difference (***p < 0.001). Confidence intervals that do not include 0 support statistical significance. [file BIP-116-e70045-s002.png]

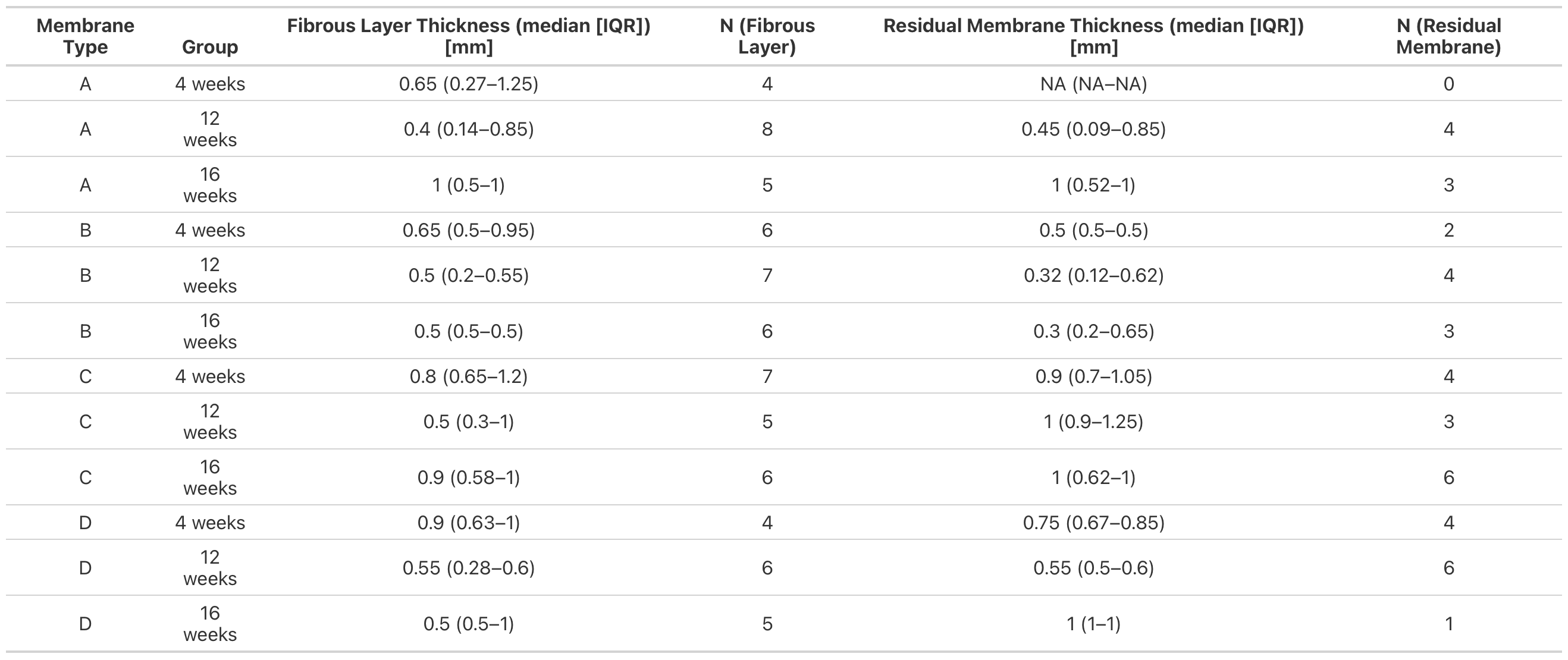

Supplement: Supplementary file 7 — Table S7: Summary of residual membrane and newly formed fibrous layer thickness. For each membrane type (A, B, C, and D), data are presented at three time points (4 weeks = 1 month, 12 weeks = 3 months, and 16 weeks = 4 months). For each time point, the table displays the median thickness (with interquartile range) and the number of observations for both the residual membrane and the surrounding fibrous layer. [file BIP-116-e70045-s004.png]
